# A Mild and Regioselective Ring-Opening of Aziridines with Acid Anhydride Using TBD or PS-TBD as a Catalyst

**DOI:** 10.3390/molecules201018482

**Published:** 2015-10-09

**Authors:** Satoru Matsukawa, Yasutaka Mouri

**Affiliations:** Department of Science Education, Faculty of Education, Ibaraki University, Ibaraki 310-8512, Japan; E-Mail: c32amg777@yahoo.co.jp

**Keywords:** organocatalyst, basecatalyst, polymercatalyst, guanidine, aziridine, amino alcohols

## Abstract

The ring-opening of *N*-tosylaziridines with various acid anhydrides catalyzed by 5 mol % of 1,5,7-triazabicyclo[4,4,0]dec-5-ene (TBD) afforded the corresponding β-amino esters in excellent yields under mild reaction conditions. Polymer-supported catalyst, PS-TBD also acts as a good catalyst for this reaction. PS-TBD was easily recovered and reused with minimal loss of activity.

## 1. Introduction

Aziridines are very useful intermediates for the synthesis of numerous nitrogen-containing biologically active compounds [1,2,3]. Therefore, nucleophilic ring-opening of aziridines by various approaches has been widely examined and developed [4,5,6,7,8,9,10]. β-amino alcohols, which can be synthesized from the nucleophilic ring-opening reactions of aziridines are useful intermediates in organic synthesis [4,7]. The ring-opening of aziridines with water is the most common method to prepare free β-amino alcohols. Many examples are reported to this day [11,12,13,14,15,16,17,18,19,20,21,22]. On the other hand, selectively protected β-amino alcohols are convenient intermediate to synthesize highly functionalized products. Therefore, the ring-opening reaction of aziridines with acid or acid anhydrides as the nucleophiles have been recently studied. To date, reactions using indium rtiflate [23], tributylphosphine [24], scandium triflate [25], ammonium-12-molybdophosphate [26], *N*-heterocyclic carbine [27] and tetrabutyl ammonium bromide [28] as catalysts have been reported. To develop of a more efficient reaction, we examined the use of TBD (1,5,7-Triazabicyclo[4,4,0]dec-5-ene) as a catalyst.

TBD is known as a superbase due to the high pKa of its conjugate acid [29,30]. Many unique reactions have been reported using TBD as an organocatalyst, such as the Henry reaction [31], Wittig and Horner-Wadsworth-Emmons reaction [32], Michael reaction [33], ring-opening polymerization [34], conjugate addition to activated alkenes [35], aminolysis of esters [36,37], intramolecular aldol reaction [38], and synthesis of pyrazolines [39], *etc.* [40,41,42,43]. Mechanistic studies of these reactions also have been discussed [44,45,46,47]. In some cases, it is considered that TBD acts as an acid-base bifunctional catalyst. Recently, we have also reported the trifluoromethylataion of aldehydes [48], and the ring-opening of aziridines with TMSCN [49] catalyzed by TBD as an organocatalyst. Herein, we report that TBD acts as an effective organobase catalyst for the ring-opening reaction of aziridines with acid anhydride.

## 2. Results and Discussion

**Figure 1:**
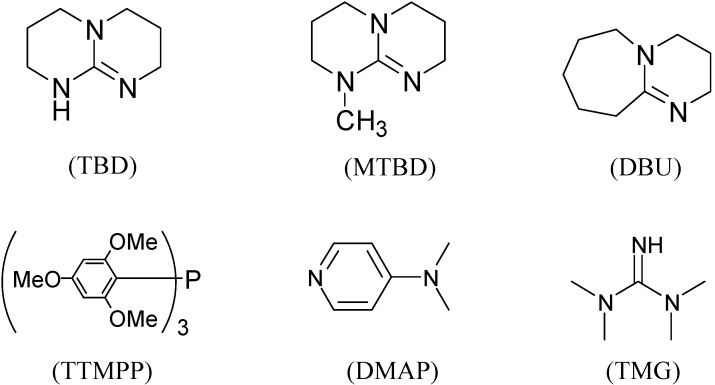
Various bases.

Initially, the ring-opening reaction of *N*-tosylaziridine **1a** with acetic anhydride was examined. The reaction was carried out by adding the **1a** and acetic anhydride in the presence of 5 mol % of TBD in DMF at 50 °C. The reaction was monitored by TLC. Hydrolytic work up with saturated NH_4_Cl at room temperature followed by flash column chromatography afforded the ring-opened products. The product was obtained at 78% yield in 24 h along with 18% of the starting material. Although the reaction was performed for 48 h, the starting material did not disappear. Then the reaction was examined at elevated temperature (80 °C), and the reaction proceeded smoothly. The desired product was obtained at 94% yield in 4 h (Table 1, entry 2). This reaction also proceeded smoothly when 2 mol % of TBD was used (Table 1, entry 3). Among the screened solvents, DMF has proved to be the most effective for this reaction (Table 1, entries 1 *vs.* 4–6). The product was obtained in lower yield when other bases, such as DBU, TMG, TTMPP and DMAP (Figure 1) were used instead of TBD (Table 1, entries 1 *vs.* 7–10). An approximately comparable yield was observed when MTBD was used as a catalyst (Table 1, entry 11).

**Table 1:** Optimization of the reaction conditions. 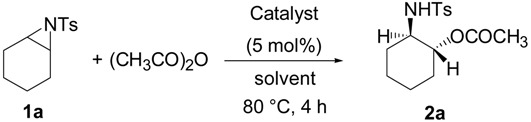

Entry	Catalyst	Solvent	Yield (%)
1	TBD	DMF ^a,b^	78
2		DMF	94
3		DMF ^c^	90
4		THF ^b,d^	45
5		MeCN ^b,e^	10
6		toluene ^b^	71
7	DBU	DMF ^b^	55
8	TMG	DMF ^b^	33
9	DMAP	DMF ^b^	34
10	TTMPP	DMF	61
11	MTBD	DMF ^b^	80

^a^ at room temperature. ^b^ in 24 h. ^c^ 2 mol % of TBD was used. ^d^ at 66 °C (reflux condition). ^e^ at 82 °C (reflux condition).

To clarify the scope of this reaction, several *N*-tosylaziridines and acid anhydrides were examined in the presence of 5 mol % TBD. In all cases, reactions were very clean and the desired products were afforded in good to excellent yields. Almost complete regioselectivity was observed when using alkyl-*N*-tosyl aziridines as substrates, and reaction on the less-substituted aziridine carbon was observed (Table 2, entries 7–10). For aryl-*N*-tosyl aziridines, in the case of a Lewis acid catalyzed reaction, selectivity demonstrated an opposite trend with alkyl-*N*-tosyl aziridines due to an electronic effect. Thus, the attack of the nucleophile at the benzylic position of aziridine occurred. However, in this Lewis base catalyzed reaction, the selectivity demonstrated the same trend: the reaction occurred on the less-substituted aziridine carbon (Table 2, entries 11–14). Reaction of propionic anhydride and benzoic anhydride also proceeded smoothly to afford the corresponding β-amino acetals in high yield. When propionic anhydride was used, regioselectivity was slightly higher than the reaction using acetic anhydride. In addition, cycloalkyl-*N*-tosyl aziridines also worked well. Unfortunately, no reaction occurred when non-acitivated aziridine such as *N*-benzylcyclohexylaziridine was employed.

Furthermore, we applied a polymer-supported TBD, PS-TBD [50,51,52,53] to this reaction. Polymer-supported catalysts have attracted significant attention in recent decades due to their inherent advantages in synthetic chemistry, e.g., simplification of reaction procedures including easy recovery of the catalyst by filtration, application to automated systems, and recycling of the catalyst [54,55,56,57,58,59]. Some unique reactions have been reported using TBD as an organocatalyst [31,60,61,62,63,64,65]. Thus, we examined the ring-opening reaction of *N*-tosylaziridine **1a** with acetic anhydride in the presence of 10 mol % of PS-TBD. As shown in Table 3, the reaction proceeded smoothly and the desired product was obtained at 85% yield in 10 h at 80 °C in DMF. A variety of *N*-tosylaziridines reacted well with acetic anhydrides to give β-amino acetates. The reaction also occurred on the less substituted aziridine carbon regardless of the type of aziridine. In addition, the recovery and reuse of PS-TBD for the reaction of *N*-tosylaziridine **1a** with acetic anhydrides also examined. After the reaction was completed, ethyl acetate was added to the reaction mixture and the catalyst was recovered by filtration. The recovered catalyst was washed, dried and then reused. The catalyst was reused, maintaining its catalytic activity after 4 uses (Table 3, entries 1–4).

**Table 2:** TBD catalyzed ring-opening of various aziridines with acid anhydrides. 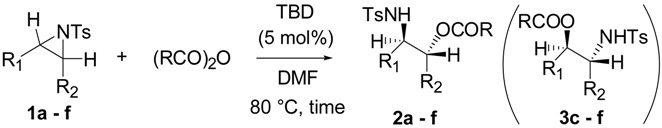

Entry	Aziridine	R	Time	Product	Yield (%) ^a^
1	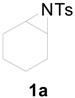	CH_3_	4 h	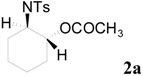	94
2		C_2_H_5_	4 h	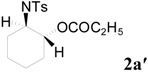	89
3		C_6_H_5_	8 h	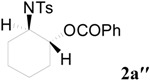	91
4 ^b^	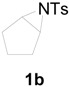	CH_3_	12 h	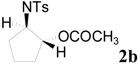	85
5		C_2_H_5_	18 h	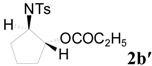	83
6	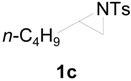	CH_3_	4 h	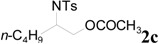	86
7		C_2_H_5_	6 h	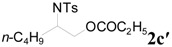	87
8		C_6_H_5_	12 h	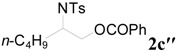	88
9	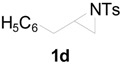	CH_3_	4 h	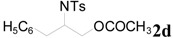	92
10		C_2_H_5_	8 h	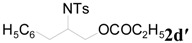	98
11	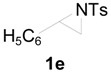	CH_3_	1 h	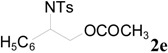	97 ^b^
12		C_2_H_5_	4 h	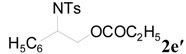	95 ^c^
13	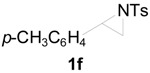	CH_3_	1 h	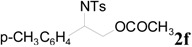	98 ^d^
14		C_2_H_5_	4 h	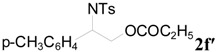	96 ^e^

^a^ Isolated yield. ^b^ Regioisomer ratio **2e**:**3e** = 85:15. ^c^ Regioisomer ratio **2eʹ**:**3eʹ** = 94:6. ^d^ Regioisomer ratio **2f**:**3f** = 87:13. ^e^ Regioisomer ratio **2fʹ**: **3fʹ** = 90:10.

**Table 3:** PS-TBD catalyzed ring-opening of various aziridines with acetic anhydride. 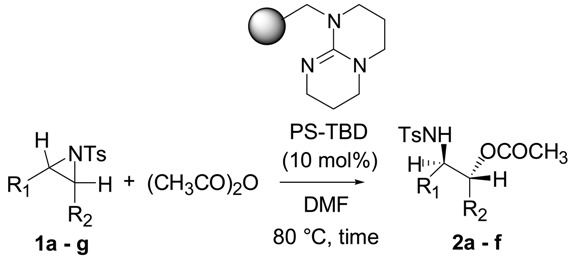

Entry	Aziridine	Product	Time	Yield (%) ^a^
1	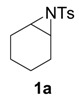	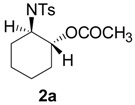	10 h	85
2			10 h	88 ^b^
3			10 h	80 ^c^
4			10 h	82 ^d^
5	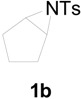	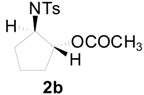	24 h	89
6	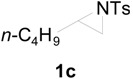	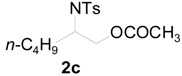	16 h	78
7	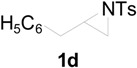	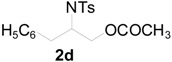	4 h	92
8	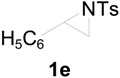	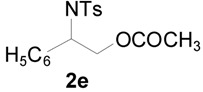	8 h	86
9	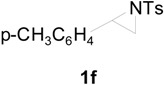	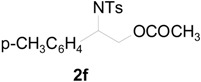	8 h	80

^a^ Isolated yield. ^b^ 2nd run. ^c^ 3rd run. ^d^ 4th run.

**Scheme 1:**
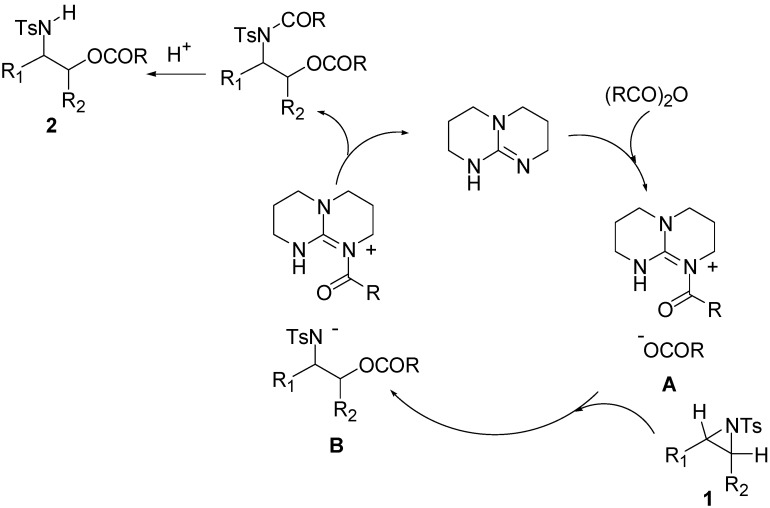
Proposed mechanism.

A possible mechanism is illustrated in Scheme 1. First, TBD activated the anhydride to form N^+^C(O)R, RC(O)O^−^ intermediate **A**. Next, this intermediate immediately reacts with aziridines to give the ring-opening product **B**. Finally, acylation occurs to give the *N*-acylated adduct with regeneration of TBD.

**Scheme 2:**
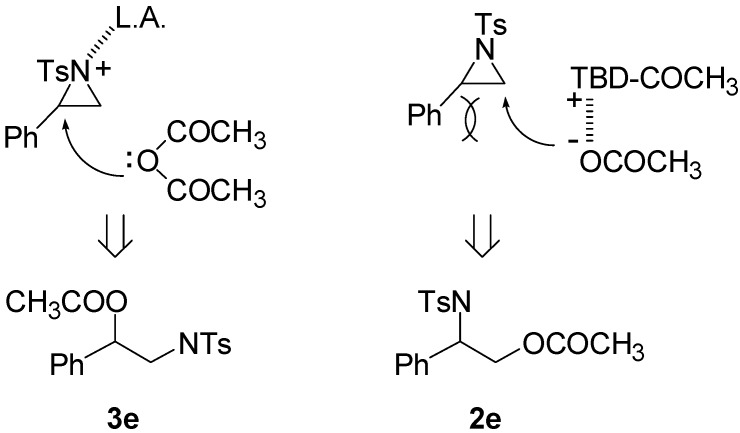
The origin of regioselectivity.

In this transition state, a steric effect has greater priority over the electronic effect (Scheme 2). Therefore, in this Lewis base catalyzed reaction, selectivity is not dependent on a substituent. The reaction occurred on the less-substituted aziridine carbon even in the case of phenyl substituted aziridine **1e**.

## 3. Experimental Section

### 3.1. General

All reactions were performed under an argon atmosphere using oven-dried glassware. Flash column chromatography was performed using silica gel Wakogel C-200 (Wako Chemical, Osaka, Japan). Preparative thin-layer chromatography was carried out on silica gel Wakogel B-5F (Wako Chemical). Dehydrate DMF, THF, toluene and CH_3_CN were purchased from Wako Chemical. Other commercially available reagent was used as received without further purification. The aziridines were prepared according to literature procedure [66]. Yields refer to isolated compounds estimated to be >95% pure, as determined by ^1^H-NMR spectroscopy. IR spectra were recorded on a JUSCO FT/IR-430 spectrometer (JASCO Corporation, Tokyo, Japan). ^1^H- and ^13^C-NMR spectra were determined for solutions in CDCl_3_ with Me_4_Si as internal standard on a Bruker Avance III instrument (Bruker Corporation, Billerica, MA, USA). HRMS data were measured on a JEOL JMS-700 mass spectrometer (JEOL Ltd., Tokyo, Japan).

### 3.2. Method

#### 3.2.1. General Procedure for TBD-Catalyzed Ring-Opening of Aziridines with Acid Anhydride

To a solution of TBD (0.05 mmol) in DMF (1 mL) was added aziridine (1.0 mmol) and acid anhydride (1.25 mmol) at room temperature. After the reaction was complete (as determinedby TLC), the reaction mixture was washed with saturated NH_4_Cl and extracted with EtOAc (2 × 10 mL). The combined organic layers were dried over Na_2_SO_4_, concentrated *in vacuo* and purified by column chromatography on silica gel (EtOAc:hexane = 1:3) to give the corresponding product.

#### 3.2.2. General Procedure for PS-TBD Catalyzed Ring-Opening of Aziridines with Acid Anhydride

To a solution of PS-TBD (0.10 mmol) in DMF (1 mL) was added aziridine (1.0 mmol) and acid anhydride (1.25 mmol) at room temperature. After the reaction was complete (as determined by TLC), EtOAc (5 mL) was added to the mixture and PS-TBD was separated by filtration. The filtrate was washed with saturated NH_4_Cl, dried over Na_2_SO_4_. The organic layer was concentrated *in vacuo* and purified by column chromatography on silica gel (EtOAc:hexane = 1:3) to give the corresponding product. The recovered catalyst is reusable after washing (acetone and water) and drying *in vacuo*.

### 3.3. General Characterization of the Products

*2-(4-Methylphenylsulfonamido)cyclohexyl acetate* (**2a**) [23]. Colorless plate; m.p. 108–110 °C; yield: 294 mg (94%); IR (KBr): 3030, 2929, 2195, 1644, 1598, 1449, 823, 700 cm^−1^; ^1^H-NMR (500 Hz, CDCl_3_) δ 1.16–1.32 (m, 4H), 1.57–1.61 (m, 1H), 1.62–1.66 (m, 1H), 1.74 (s, 3H), 1.85–1.89 (m, 1H), 1.96–2.00 (m, 1H), 2.38 (s, 3H), 3.12–3.18 (m, 1H), 4.53 (dt, *J* = 4.6, 10.4 Hz, 1H), 4.95 (d, *J* = 6.8 Hz, 1H), 7.25 (d, *J* = 8.0 Hz, 2H), 7.71 (d, *J* = 8.0 Hz, 2H); ^13^C-NMR (125 MHz, CDCl_3_) δ 20.9, 21.5, 23.6, 24.2, 31.1, 33.5, 56.9, 74.1, 126.9, 129.6, 138.7, 143.0, 171.4; HRMS (FAB): *m*/*z*: cald for C_15_H_22_NO_4_S: 312.1270; found: 312.1280 [M + H]^+^.

*2-(4-Methylphenylsulfonamido)cyclohexyl propionate* (**2aʹ**) [23]. Colorless cube; m.p. 92–94 °C; yield: 289 mg (89%); IR (KBr): 3284, 2957, 1736, 1460, 1331, 1162, 1092, 815, 666 cm^−1^; ^1^H-NMR (500 Hz, CDCl_3_) δ 1.02 (t, *J* = 7.5 Hz, 3H), 1.20–1.39 (m, 4H), 1.62–1.67 (m, 1H), 1.68–1.71 (m, 1H), 1.95–1.99 (m, 1H), 2.00–2.05 (m, 1H), 2.07 (q, *J* = 7.5 Hz, 2H), 2.42 (s, 3H), 3.19-3.24 (m, 1H), 4.59 (dq, *J* = 3.4, 10.4 Hz, 1H), 5.04 (d, *J* = 10.4 Hz, 1H), 7.29 (d, *J* = 8.6 Hz, 2H), 7.74 (d, *J* = 8.6 Hz, 2H); ^13^C-NMR (125 MHz, CDCl_3_) δ 8.8, 21.4, 23.6, 24.1, 27.4, 31.0, 33.3, 56.9, 73.8, 126.8, 129.5, 138.7, 142.9, 174.8; HRMS (FAB): *m*/*z*: cald for C_16_H_24_NO_4_S: 326.1426; found: 326.1410 [M + H]^+^.

*2-(4-Methylphenylsulfonamido)-1-phenylcarbonyloxyclohexane* (**2aʹʹ**) [24]. White solid; m.p. 128–130 °C; yield: 340 mg (91%); IR (KBr): 3321, 2926, 1786, 1702, 1598, 1450, 1320, 1212, 1155, 1014, 715, 666 cm^−1^; ^1^H-NMR (500 Hz, CDCl_3_) δ 1.20–1.28 (m, 2H), 1.27–1.42 (m, 2H), 1.64–1.66 (m, 1H), 1.67–1.71 (m, 1H), 1.95–2.01 (m, 1H), 2.10–2.17 (m, 1H), 2.15 (s, 3H), 3.26–3.32 (m, 1H), 4.79 (dt, *J* = 4.7, 10.7 Hz, 1H), 5.16 (d, *J* = 7.4 Hz, 1H), 6.88 (d, *J* = 8.0 Hz, 2H), 7.32 (d, *J* = 8.0 Hz, 2H), 7.50 (d, *J* = 6.4 Hz, 1H), 7.55 (d, *J* = 8.2 Hz, 2H), 7.74 (d, *J* = 7.0 Hz, 2H); ^13^C-NMR (125 MHz, CDCl_3_) δ 21.2, 23.6, 24.1, 31.1, 33.8, 57.1, 74.5, 126.4, 128.0, 128.8, 129.3, 129.6, 130.4, 132.8, 138.0, 142.5, 166.6; HRMS (FAB): *m*/*z*: cald for C_20_H_24_NO_4_S: 374.1426; found: 374.1420 [M + H]^+^.

*2-(4-Methylphenylsulfonamido)cyclopentyl acetate* (**2b**) [23]. Colorless oil; yield: 253 mg (85%); IR (neat): 3278, 2925, 1732, 1455, 1326, 1158, 1092, 815, 666 cm^−1^; ^1^H-NMR (500 Hz, CDCl_3_) δ 1.44–1.50 (m, 2H), 1.57–1.64 (m, 2H), 1.84 (s, 3H), 1.95–1.99 (m, 2H), 2.39 (s, 3H), 3.40–3.44 (m, 1H), 4.82 (dt, *J* = 5.5, 7.5 Hz, 1H), 5.33–5.39 (brs, 1H), 7.26 (d, *J* = 8.1 Hz, 2H), 7.72 (d, *J* = 8.1 Hz, 2H); ^13^C-NMR (125 MHz, CDCl_3_) δ 20.7, 20.8, 21.4, 29.3, 31.0, 59.7, 79.5, 127.1, 129.5, 137.3, 143.3, 171.0; HRMS (FAB): *m*/*z*: cald for C_14_H_20_NO_4_S: 298.1113; found: 298.1118 [M + H]^+^.

*2-(4-Methylphenylsulfonamido)cyclopentyl propionate* (**2bʹ**) [25]. Colorless oil; yield: 258 mg (83%); IR (neat): 3276, 2941, 1741, 1462, 1356, 1165, 813, 666 cm^−1^; ^1^H-NMR (500 Hz, CDCl_3_) δ 0.94 (t, *J* = 7.6 Hz, 3H), 1.40–1.45 (m, 2H), 1.50–1.57 (m, 2H), 1.85–1.95 (m, 2H), 2.00–2.10 (m, 2H), 2.32 (s, 3H), 3.33–3.40 (m, 1H), 4.80 (dt, *J* = 5.4, 7.4 Hz, 1H), 5.70 (d, *J* = 7.4 Hz, 1H), 7.21 (d, *J* = 8.1 Hz, 2H), 7.68 (d, *J* = 8.1 Hz, 2H); ^13^C-NMR (125 MHz, CDCl_3_) δ 8.7, 20.6, 27.2, 29.2, 30.8, 59.4, 79.2, 126.9, 129.3, 137.3, 143.1, 174.2; HRMS (FAB): *m*/*z*: cald for C_15_H_22_NO_4_S: 312.1270; found: 312.1285 [M + H]^+^.

*2-(4-Methylphenylsulfonamido)hexyl acetate* (**2c**) [23]. Colorless oil; yield: 269 mg (86%); IR (neat): 3281, 2957, 2871, 1741, 1598, 1431, 1329, 1239, 1162, 1093, 815, 666 cm^−1^; ^1^H-NMR (500 Hz, CDCl_3_) δ 0.76 (t, *J* = 7.2 Hz, 3H), 1.06–1.30 (m, 4H), 1.32–1.45 (m, 2H), 1.90 (s, 3H), 2.39 (s, 3H), 3.38–3.43 (m, 1H), 3.85 (dd, *J* = 4.3, 11.4 Hz, 1H), 3.94 (dd, *J* = 5.4, 11.4 Hz, 1H), 4.90 (d, *J* = 8.5 Hz, 1H), 7.26 (d, *J* = 7.9 Hz, 2H), 7.73 (d, *J* = 7.9 Hz, 2H); ^13^C-NMR (125 MHz, CDCl_3_) δ 13.7, 20.6, 21.4, 22.2, 27.4, 32.0, 52.8, 65.7, 127.0, 129.6, 138.1, 143.3, 170.8; HRMS (FAB): *m*/*z*: cald for C_15_H_24_NO_4_S: 314.1426; found: 314.1409 [M + H]^+^.

*2-(4-Methylphenylsulfonamido)hexyl propionate* (**2cʹ**) [26]. Colorless oil; yield: 285 mg (87%); IR (neat): 3283, 2957, 1740, 1462, 1330, 1162, 1092, 815, 667 cm^−1^; ^1^H-NMR (500 Hz, CDCl_3_) δ 0.76 (t, *J* = 7.1 Hz, 3H), 1.05 (t, *J* = 7.6 Hz, 3H), 1.15–1.25 (m, 4H), 1.35–1.48 (m, 2H), 2.18 (q, *J* = 7.6 Hz, 2H), 2.39 (s, 3H), 3.36-3.45 (m, 1H), 3.86 (dd, *J* = 4.3, 11.4 Hz, 1H), 3.96 (dd, *J* = 5.5, 11.4 Hz, 1H), 4.77–4.82 (m, 1H), 7.26 (d, *J* = 8.4 Hz, 2H), 7.73 (d, *J* = 8.4 Hz, 2H); ^13^C-NMR (125 MHz, CDCl_3_) δ 8.9, 13.7, 21.4, 22.2, 27.1, 27.4, 32.0, 52.9, 65.6, 126.9, 129.6, 138.1, 143.3, 174.2; HRMS (FAB): *m*/*z*: cald for C_16_H_26_NO_4_S: 328.1583; found: 328.1596 [M + H]^+^.

*2-(4-Methylphenylsulfonamido)-1-phenylcarbonyloxyhexane* (**2cʹʹ**) [23]. White solid; m.p. 65–69 °C; yield: 330 mg (88%); IR (KBr): 3288, 2929, 1787, 1560, 1452, 1273, 1162, 815, 708, 615 cm^−1^; ^1^H-NMR (500 Hz, CDCl_3_) δ 0.78 (t, *J* = 7.2 Hz, 3H), 1.13–1.31 (m, 4H), 1.45–1.59 (m, 2H), 2.29 (s, 3H), 3.53–3.62 (m, 1H), 4.11 (dd, *J* = 4.2, 11.5 Hz, 1H), 4.22 (dd, *J* = 5.6, 11.5 Hz, 1H), 5.16 (d, *J* = 8.3 Hz, 1H), 7.11 (d, *J* = 8.0 Hz, 2H), 7.36 (t, *J* = 7.5 Hz, 2H), 7.52 (t, *J* = 7.5 Hz, 1H), 7.71 (d, *J* = 8.3 Hz, 2H), 7.89 (d, *J* = 7.1 Hz, 2H); ^13^C-NMR (125 MHz, CDCl_3_) δ 13.7, 21.4, 22.2, 27.5, 32.2, 53.0, 66.2, 126.8, 128.2, 129.6, 133.1, 138.0, 143.2, 166.2; HRMS (FAB): *m*/*z*: cald for C_20_H_26_NO_4_S: 376.1583; found: 374.1564 [M + H]^+^.

*2-(4-Methylphenylsulfonamido)-3-phenylpropyl acetate* (**2d**) [23]. White solid; m.p. 67–69 °C; yield: 320 mg (92%); IR (KBr): 3280, 2953, 1742, 1455, 1329, 1160, 1092, 815, 667 cm^−1^; ^1^H-NMR (500 Hz, CDCl_3_) δ 1.93 (s, 3H), 2.37 (s, 3H), 2.75 (d, *J* = 7.0 Hz, 2H), 3.62–3.69 (m, 1H), 3.90 (dd, *J* = 5.3, 15.0 Hz, 1H), 3.92 (dd, *J* = 5.4, 15.0 Hz, 1H), 5.18 (d, *J* = 8.1 Hz, 1H), 7.00 (d, *J* = 8.0 Hz, 2H), 7.14–7.22 (m, 5H), 7.62 (d, *J* = 8.2 Hz, 2H); ^13^C-NMR (125 MHz, CDCl_3_) δ 20.6, 21.3, 38.5, 53.8, 64.8, 126.7, 126.8, 128.6, 129.1, 129.5, 136.2, 137.5, 143.2, 170.7; HRMS (FAB): *m*/*z*: cald for C_18_H_22_NO_4_S: 348.1270; found: 348.1254 [M + H]^+^.

*2-(4-Methylphenylsulfonamido)-3-phenylpropyl propionate* (**2dʹ**) [25]. Colorless oil; yield: 354 mg (98%); IR (neat): 3281, 2942, 1740, 1456, 1330, 1160, 1092, 814, 666 cm^−1^; ^1^H-NMR (500 Hz, CDCl_3_) δ 1.08 (t, *J* = 7.6 Hz, 3H), 2.20 (q, *J* = 7.6 Hz, 2H), 2.40 (s, 3H), 3.34-3.41 (m, 1H), 3.92 (dd, *J* = 4.7, 11.5 Hz, 1H), 3.97 (dd, *J* = 5.5, 11.5 Hz, 1H), 4.84 (d, *J* = 7.9 Hz, 1H), 7.02 (d, *J* = 7.3Hz, 2H), 7.16–7.23 (m, 5H), 7.63 (d, *J* = 8.3 Hz, 2H); ^13^C-NMR (125 MHz, CDCl_3_) δ 8.9, 21.4, 27.2, 38.5, 53.9, 64.7, 126.8, 126.9, 128.7, 129.2, 129.6, 136.2, 137.6, 143.3, 174.1; HRMS (FAB): *m*/*z*: cald for C_19_H_24_NO_4_S: 362.1426; found: 362.1445 [M + H]^+^.

*2-(4-Methylphenylsulfonamido)-2-phenylethyl acetate* (**2e**) [23]. Colorless oil; yield: 300 mg (90%); IR (neat): 3280, 2923, 1742, 1434, 1328, 1160, 1092, 814, 666 cm^−1^; ^1^H-NMR (500 Hz, CDCl_3_) δ 1.91 (s, 3H), 2.35 (s, 3H), 4.14 (dd, *J* = 4.9, 11.6 Hz, 1H), 4.18 (dd, *J* = 7.9, 11.6 Hz, 1H), 4.60 (dt, *J* = 4.9, 7.3 Hz, 1H), 5.43 (d, *J* = 7.0 Hz, 1H), 7.08–7.12 (m, 4H), 7.16–7.18 (m, 2H), 7.26–7.30 (m, 1H), 7.57 (d, *J* = 8.4 Hz, 2H); ^13^C-NMR (125 MHz, CDCl_3_) δ 20.6, 21.3, 56.9, 66.5, 126.8, 127.1, 128.6, 129.4, 137.0, 137.5, 143.3, 170.8; HRMS (FAB): *m*/*z*: cald for C_17_H_20_NO_4_S: 334.1113; found: 334.1119 [M + H]^+^.

*2-(4-Methylphenylsulfonamido)-2-phenylethyl propionate* (**2eʹ**) [25]. Colorless oil; yield: 306 mg (88%); IR (neat): 3280, 2924, 1741, 1516, 1438, 1160, 1090, 813, 666 cm^−1^; ^1^H-NMR (500 Hz, CDCl_3_) δ 1.02 (t, *J* = 7.6 Hz, 3H), 2.18 (q, *J* = 7.6 Hz, 2H), 2.35 (s, 3H), 4.17 (dd, *J* =5.0 Hz, 11.6 Hz, 1H), 4.20 (dd, *J* = 7.4, 11.6 Hz, 1H), 4.62 (dt, *J* = 4.9, 7.3 Hz, 1H), 5.72 (d, 6.6 Hz, 1H), 7.12–7.15 (m, 4H), 7.17–7.19 (m, 2H), 7.27–7.30 (m, 1H), 7.59 (d, *J* = 8.3 Hz, 2H); ^13^C-NMR (125 MHz, CDCl_3_) δ 8.8, 21.3, 27.1, 56.9, 66.3, 126.8, 127.0, 128.5, 129.3, 137.1, 137.4, 143.1, 174.2; HRMS (FAB): *m*/*z*: cald for C_18_H_22_NO_4_S: 348.1270; found: 348.1262 [M + H]^+^.

*2-(4-Methylphenylsulfonamido)-2-(4-methylphenyl)ethyl acetate* (**2f**) [23]. White solid; m.p. 88–90 °C; yield: 316 mg (91%); IR (neat): 3278, 2925, 1742, 1495, 1330, 1161, 1044, 815, 668 cm^−1^; ^1^H-NMR (500 Hz, CDCl_3_) δ 1.93 (s, 3H), 2.28 (s, 3H), 2.38 (s, 3H), 4.14 (dd, *J* = 4.8, 11.6 Hz, 1H), 4.19 (dd, *J* = 7.6, 11.6 Hz, 1H), 4.56 (dt, *J* = 4.9, 7.3 Hz, 1H), 5.66 (dd, *J* = 5.0, 7.3 Hz, 1H), 7.00–7.02 (m, 4H), 7.17 (d, *J* = 8.0 Hz, 2H), 7.60 (d, *J* = 8.0 Hz, 2H); ^13^C-NMR (125 MHz, CDCl_3_) δ 20.6, 21.0, 21.4, 56.5, 66.4, 126.7, 127.0, 129.2, 129.3, 133.9, 137.4, 137.8, 143.2, 170.8; HRMS (FAB): *m*/*z*: cald for C_18_H_22_NO_4_S: 348.1270; found: 348.1288 [M + H]^+^.

*2-(4-Methylphenylsulfonamido)-2-(4-methylphenyl)ethyl acetate* (**2fʹ**) [25]. White solid; m.p. 98–100 °C; yield: 310 mg (83%); IR (neat): 3283, 2982, 1740, 1598, 1462, 1348, 1161, 1088, 814, 666 cm^−1^; ^1^H-NMR (500 Hz, CDCl_3_) δ 1.04 (t, *J* = 7.6 Hz, 3H), 2.20 (q, *J* = 7.6 Hz, 2H), 2.28 (s, 3H), 2.38 (s, 3H), 4.15 (dd, *J* =4.8, 11.6 Hz, 1H), 4.20 (dd, *J* = 7.6, 11.6 Hz, 1H), 4.57 (dt, *J* = 4.8, 7.2 Hz, 1H), 5.22 (d, *J* = 6.8 Hz, 1H), 7.00–7.02 (m, 4H), 7.17 (d, *J* = 8.0 Hz, 2H), 7.59 (d, *J* = 8.0 Hz, 2H); ^13^C-NMR (125 MHz, CDCl_3_) δ 8.8, 21.0, 21.4, 56.7, 66.3, 126.7, 127.0, 129.2, 129.4, 134.0, 137.4, 137.9, 143.2, 174.3; HRMS (FAB): *m*/*z*: cald for C_19_H_24_NO_4_S: 362.1426; found: 362.1415 [M + H]^+^.

Copies of ^1^H- and ^13^C-NMR Spectra of products **2a**–**2f**, **2aʹ**–**2fʹ**, **2aʹʹ**, and **2cʹʹ** could be found in the supplementary materials.

## 4. Conclusions

In conclusion, we have demonstrated TBD catalyzed ring-opening reactions of *N*-tosylaziridine with acid anhydrides. A broad range of *N*-tosylaziridine and acid anhydrides could be applied using 5 mol % TBD. Furthermore, polymer-supported catalyst, PS-TBD also act as a good catalyst for this reaction. PS-TBD was easily recovered and reused with minimal loss of activity. These reactions provide a simple and convenient method for the synthesis of highly functionalized β-amino alcohols.

## Supplementary Materials

Supplementary materials can be accessed at: http://www.mdpi.com/1420-3049/20/10/18482/s1.
