# Supplementary material for: A Mild and Regioselective Ring-Opening of Aziridines with Acid Anhydride Using TBD or PS-TBD as a Catalyst
Source: Molecules. 2015 Oct 9;20(10):18482–95. doi: 10.3390/molecules201018482 (PMC6332191; doi:10.3390/molecules201018482)
Supplement: Supplementary file 1 [file molecules-20-18482-s001.pdf]

Supplementary Material

Instrumentation and Chemicals **S1**
Experimental Procedure **S1**
Copies of ^{1}H- and ^{13}C-NMR Spectra of Products **S2**

1. Instrumentation and Chemicals

All reactions were performed under an argon atmosphere using oven-dried glassware. Flash column chromatography was performed using silica gel Wakogel C-200 (Wako Chemical, Osaka, Japan). Preparative thin-layer chromatography was carried out on silica gel Wakogel B-5F (Wako Chemical). Dehydrate DMF, THF, toluene and CH_3CN were purchased from Wako Chemical. Other commercially available reagent was used as received without further purification. The aziridines were prepared according to literature procedure [66]. Yields refer to isolated compounds estimated to be >95% pure, as determined by ^{1}H-NMR spectroscopy. IR spectra were recorded on a JUSCO FT/ IR-430 spectrometer (JASCO Corporation, Tokyo, Japan). ^{1}H- and ^{13}C-NMR spectra were determined for solutions in $CDCl_3$ with Me_4Si as internal standard on a Bruker Avance III instrument (Bruker Corporation, Billerica, MA, USA). HRMS data were measured on a JEOL JMS-700 mass spectrometer (JEOL Ltd., Tokyo, Japan).

2. General Procedure for TBD-Catalyzed Ring-Opening of Aziridines with Acid Anhydride

To a solution of TBD (0.05 mmol) in DMF (1 mL) was added aziridine (1.0 mmol) and acid anhydride (1.25 mmol) at room temperature. After the reaction was complete (as determined by TLC), the reaction mixture was treated with saturated NH_4Cl and extracted with EtOAc (2 × 10 mL). The combined organic layers were dried over Na_2SO_4, concentrated *in vacuo* and purified by column chromatography on silica gel (EtOAc:hexane = 1:3) to give the corresponding product.

3. General Procedure for PS-TBD Catalyzed Ring-Opening of Aziridines with Acid Anhydride

To a solution of PS-TBD (0.10 mmol) in DMF (1 mL) was added aziridine (1.0 mmol) and acid anhydride (1.25 mmol) at room temperature. After the reaction was complete (as determined by TLC), EtOAc (5 mL) was added to the mixture and PS-TBD was separated by filtration. The filtrate was washed with saturated NH_4Cl, dried over Na_2SO_4, concentrated *in vacuo* and purified by column chromatography on silica gel (EtOAc:hexane = 1:3) to give the corresponding product. The recovered catalyst is reusable after washing (acetone and water) and drying *in vacuo*.

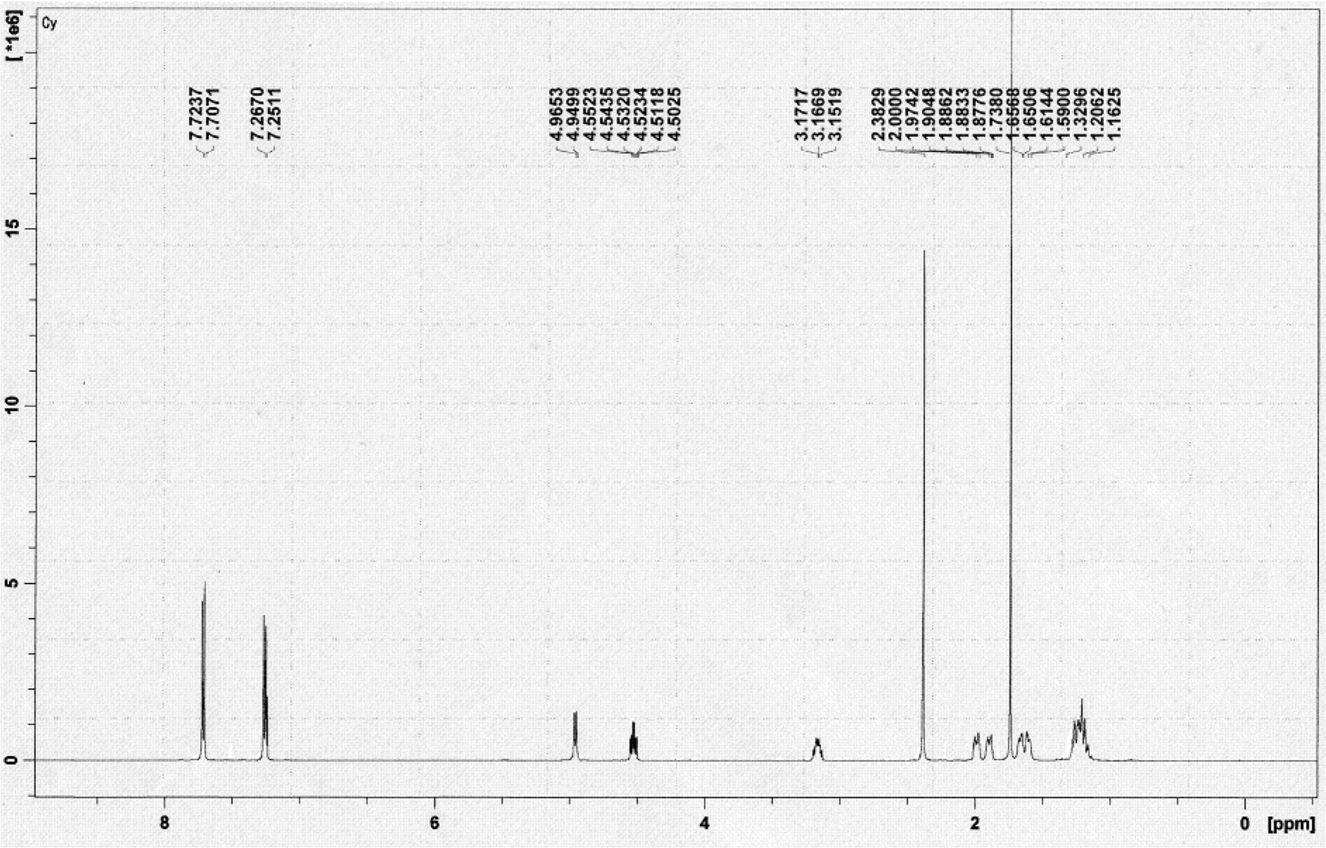

Figure S1. ^{1}H-NMR Spectrum of compound **2a**.

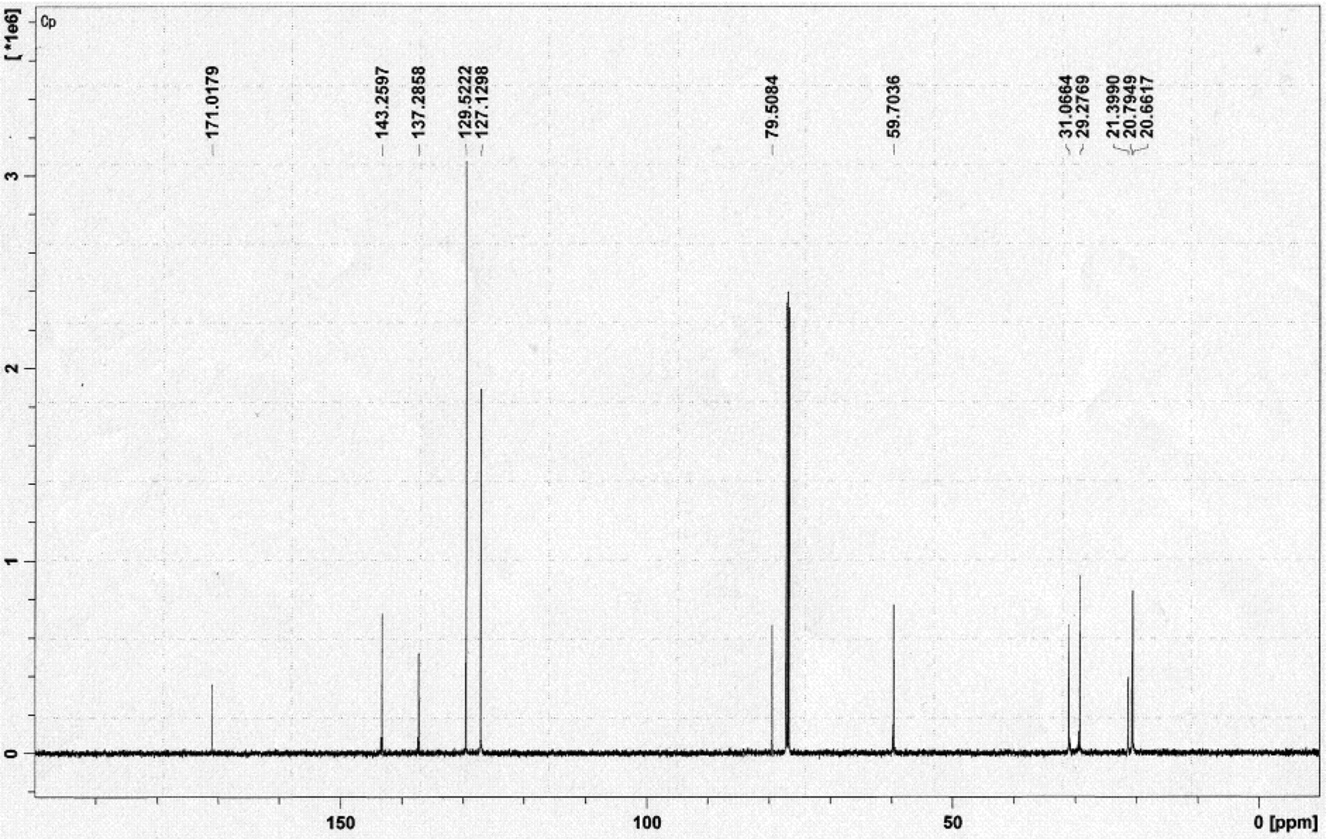

Figure S2. ^{13}C-NMR Spectrum of compound **2a**.

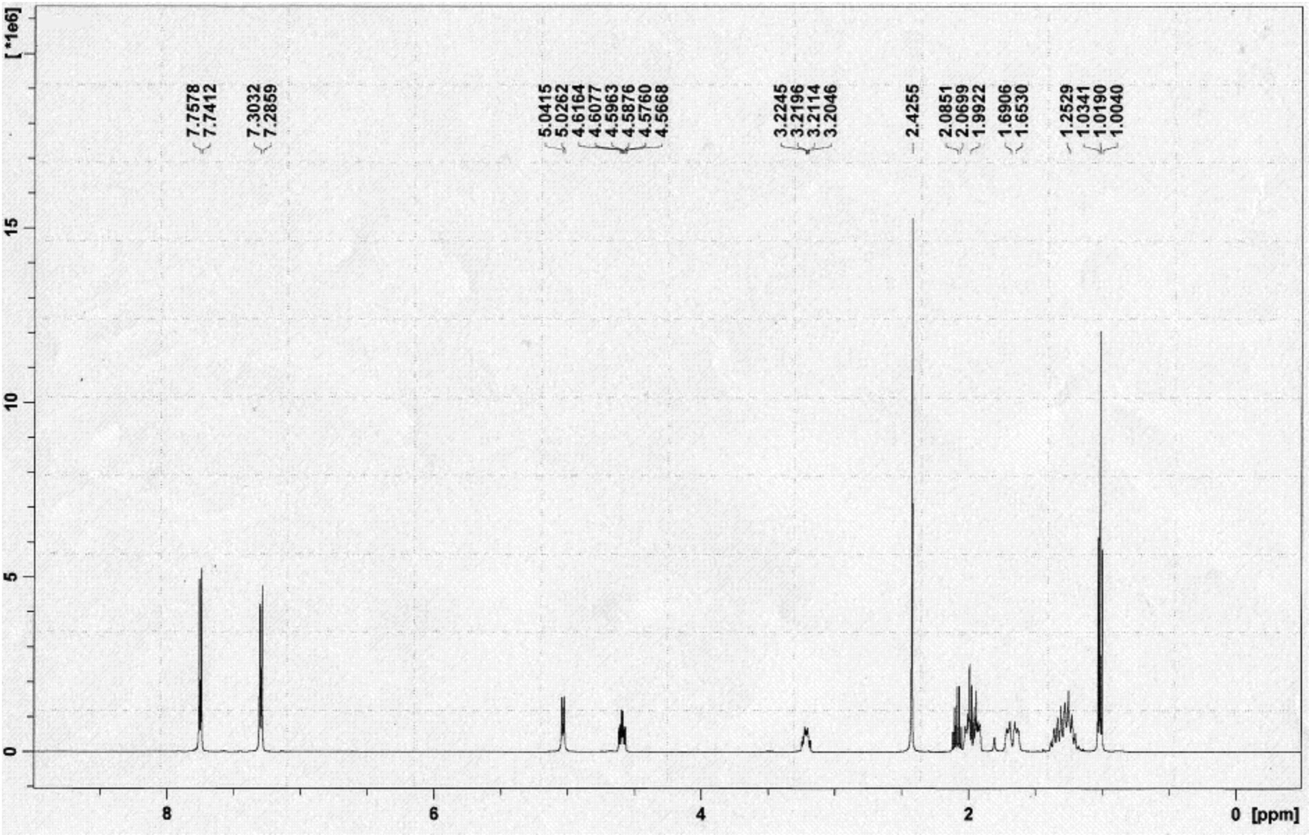

Figure S3. ^{1}H-NMR Spectrum of compound **2a′**.

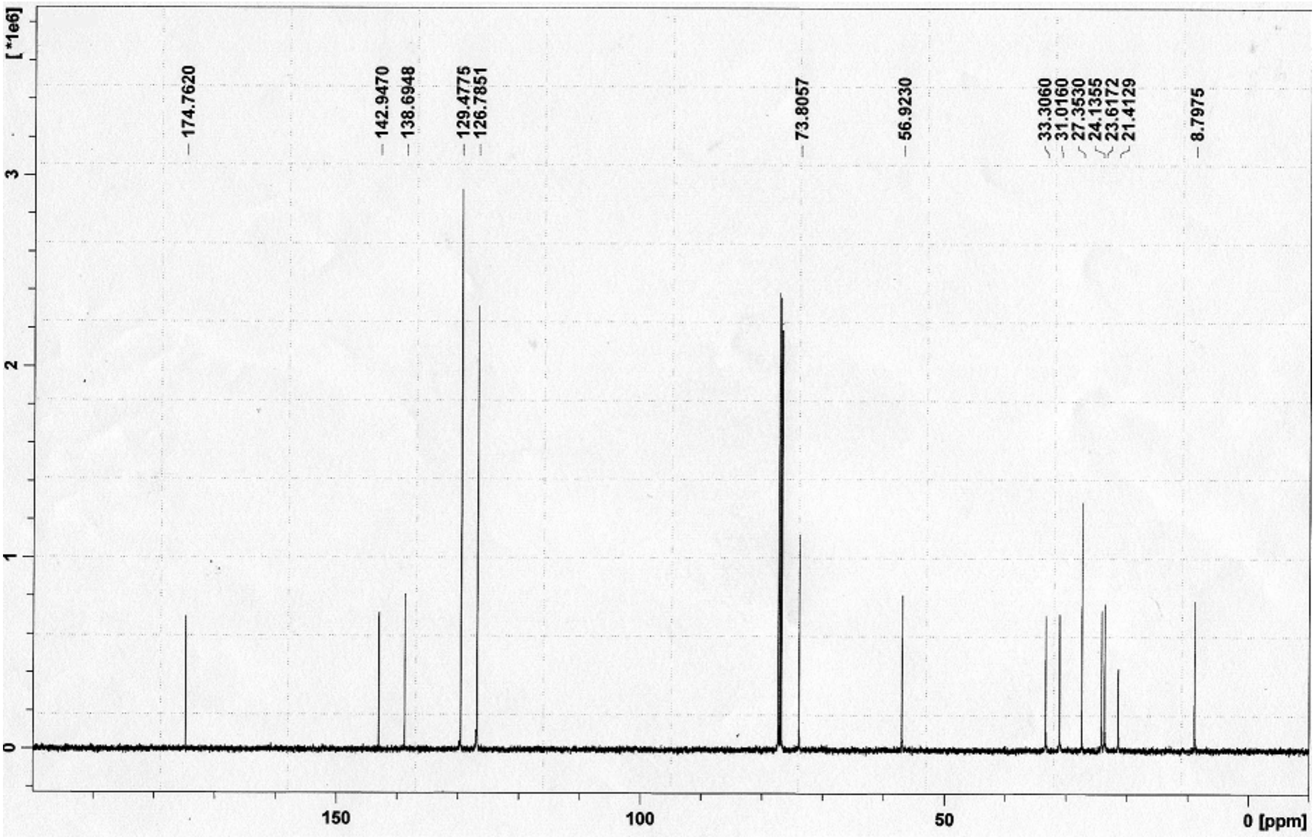

Figure S4. ^{13}C-NMR Spectrum of compound **2a′**.

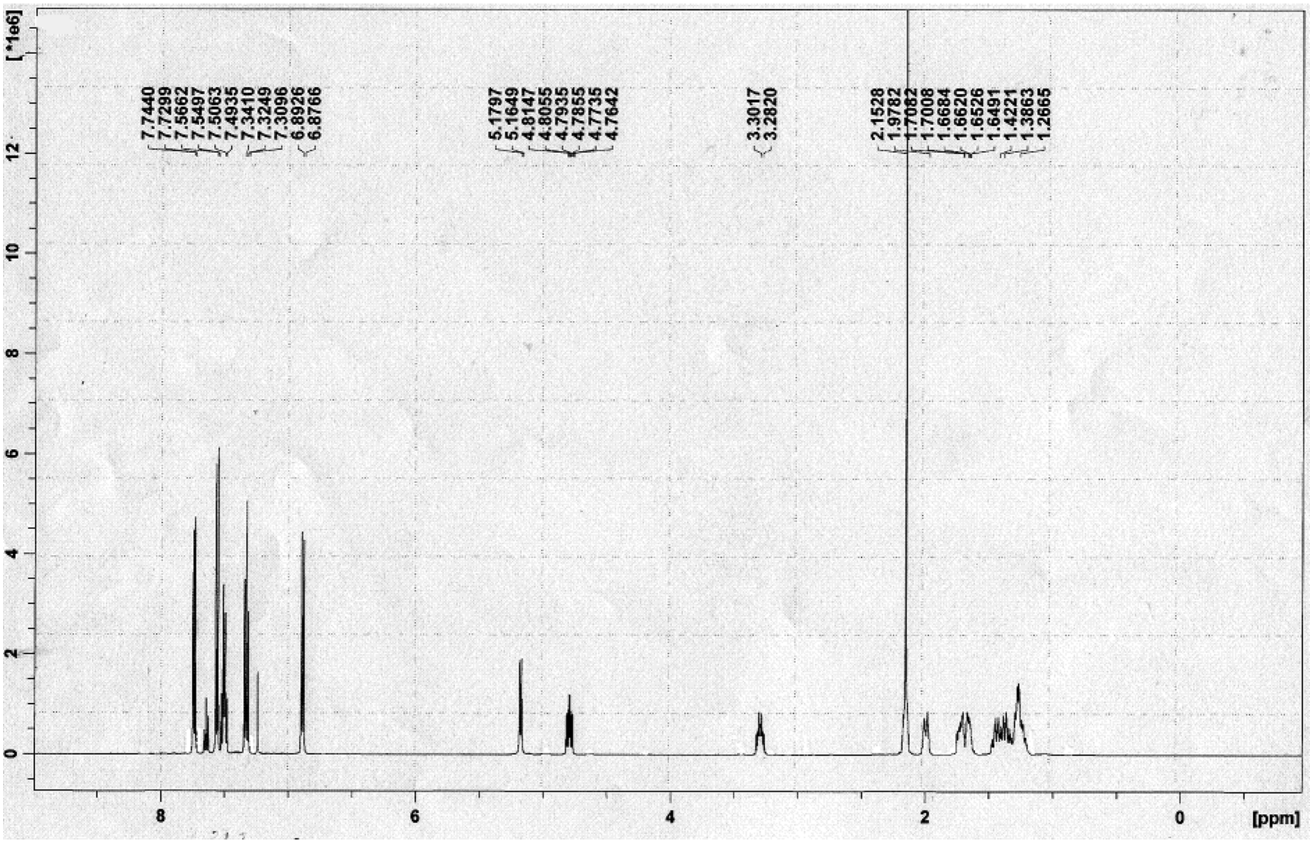

Figure S5. ^{1}H-NMR Spectrum of compound **2a″**.

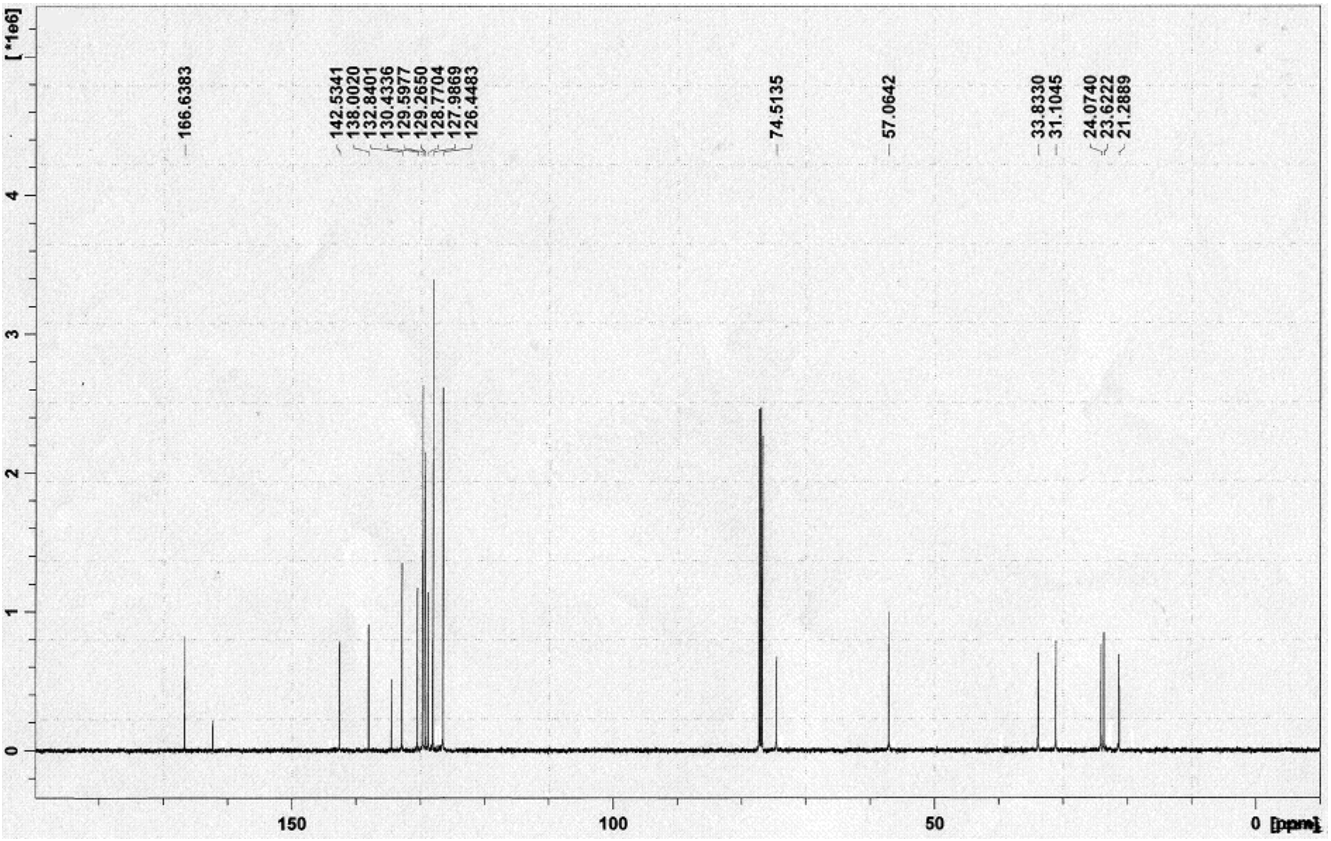

Figure S6. ^{13}C-NMR Spectrum of compound **2a′**.

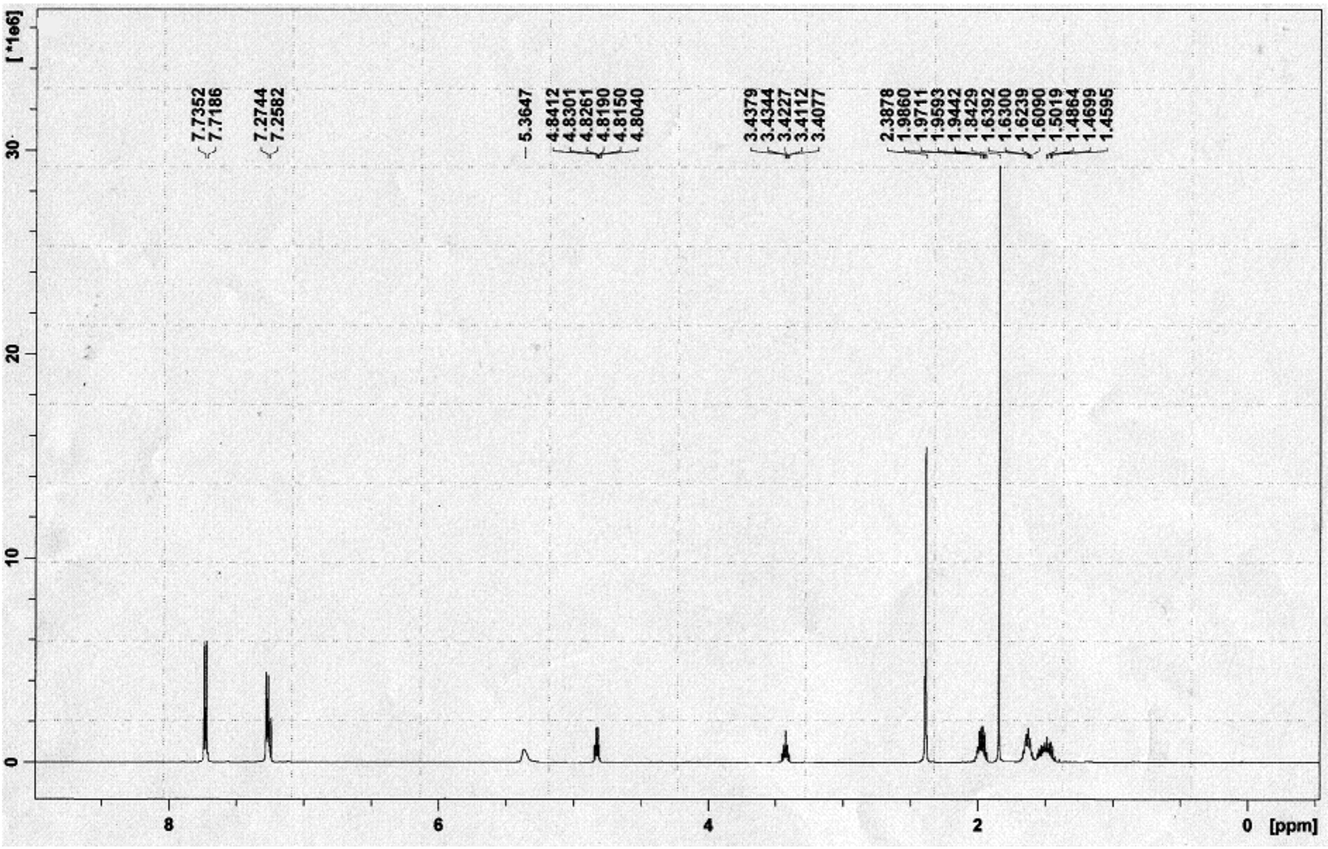

Figure S7. ^{1}H-NMR Spectrum of compound **2b**.

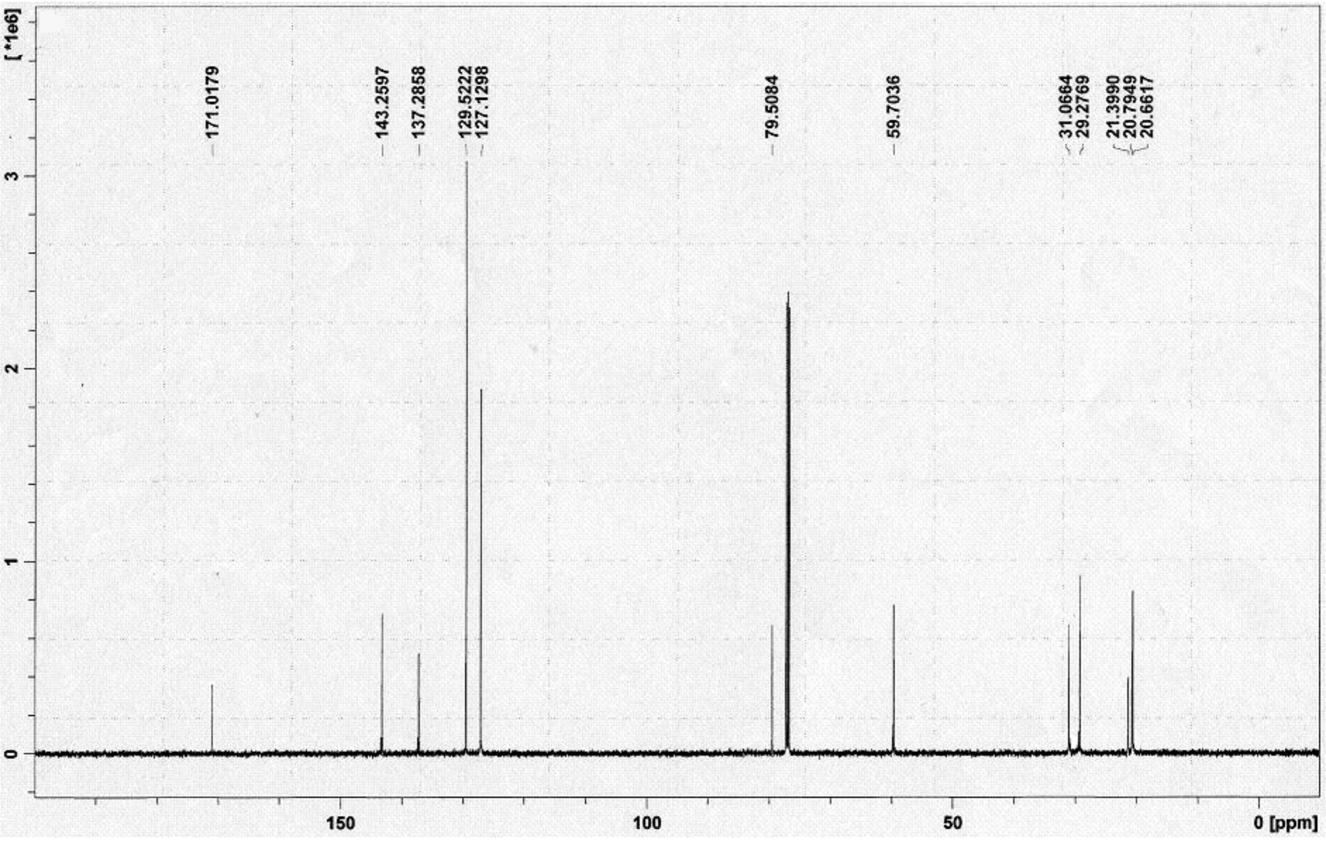

Figure S8. ^{13}C-NMR Spectrum of compound **2b**.

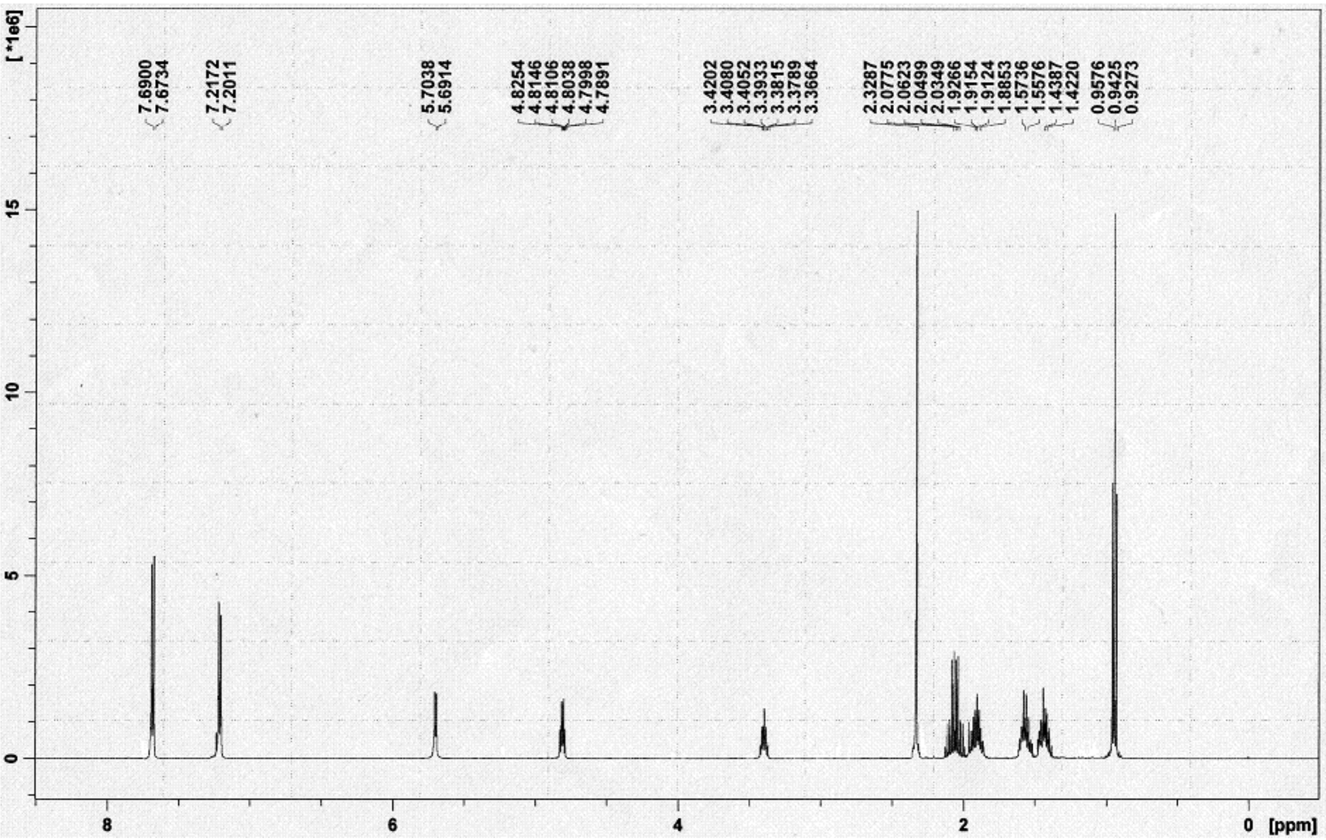

Figure S9. ^{1}H-NMR Spectrum of compound **2b′**.

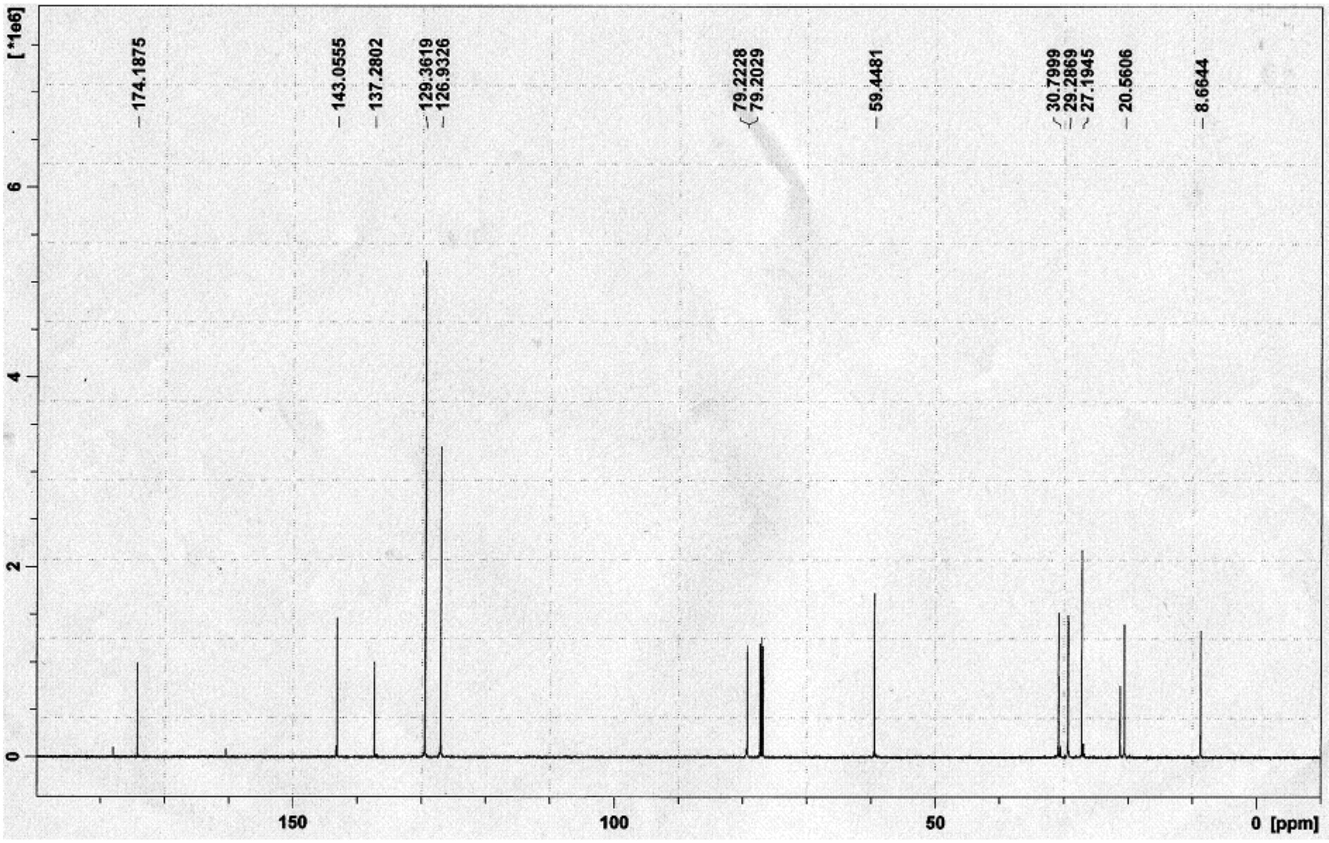

Figure S10. ^{13}C-NMR Spectrum of compound **2b′**.

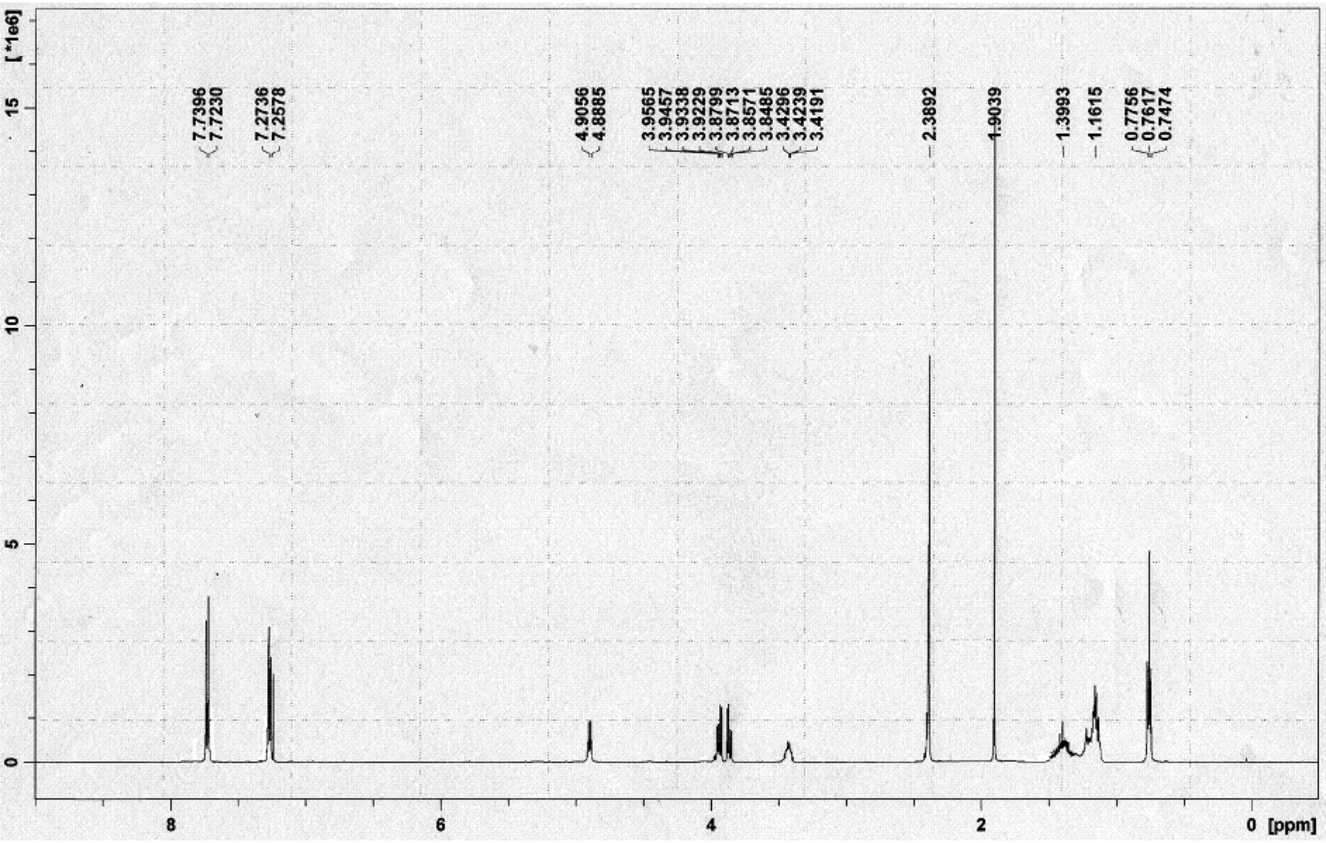

Figure S11. ^{1}H-NMR Spectrum of compound **2c**.

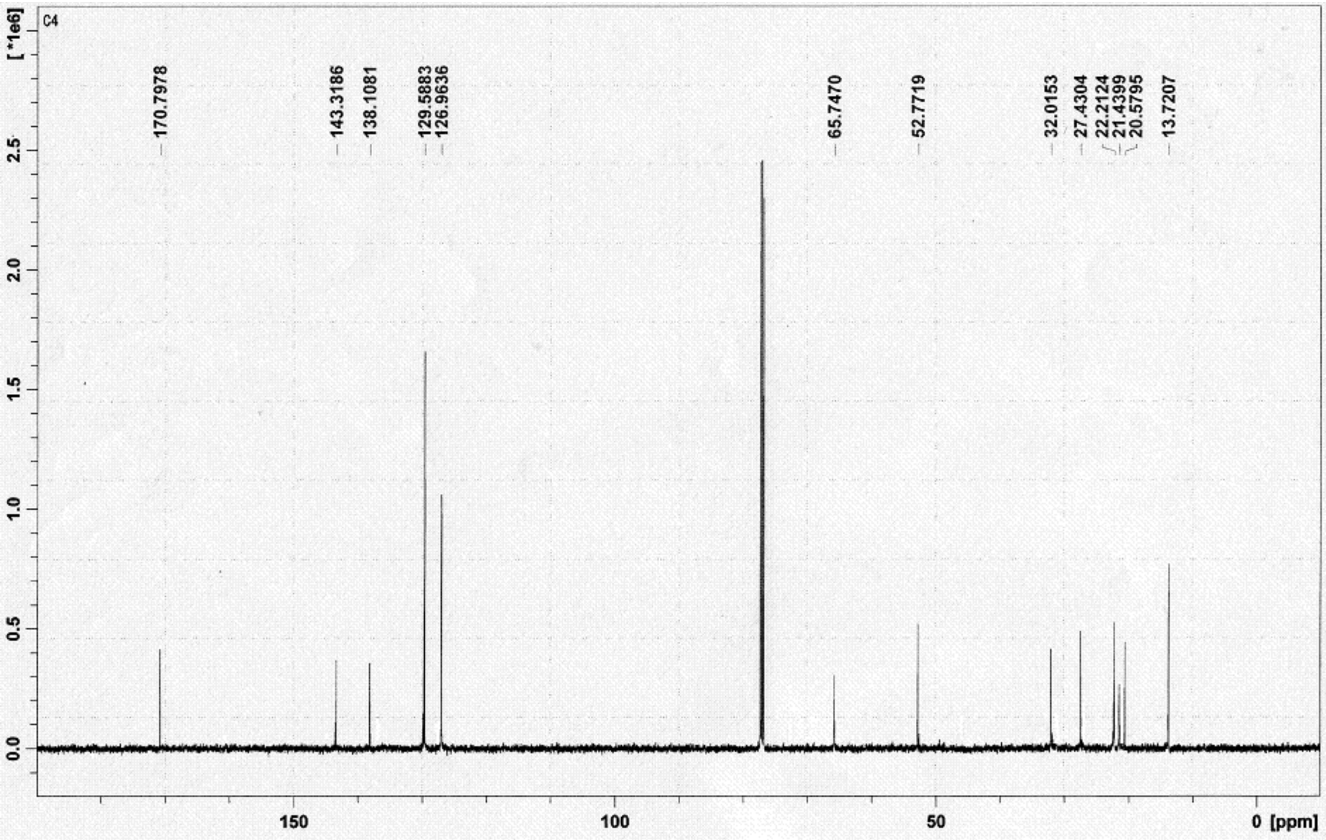

Figure S12. ^{13}C-NMR Spectrum of compound **2c**.

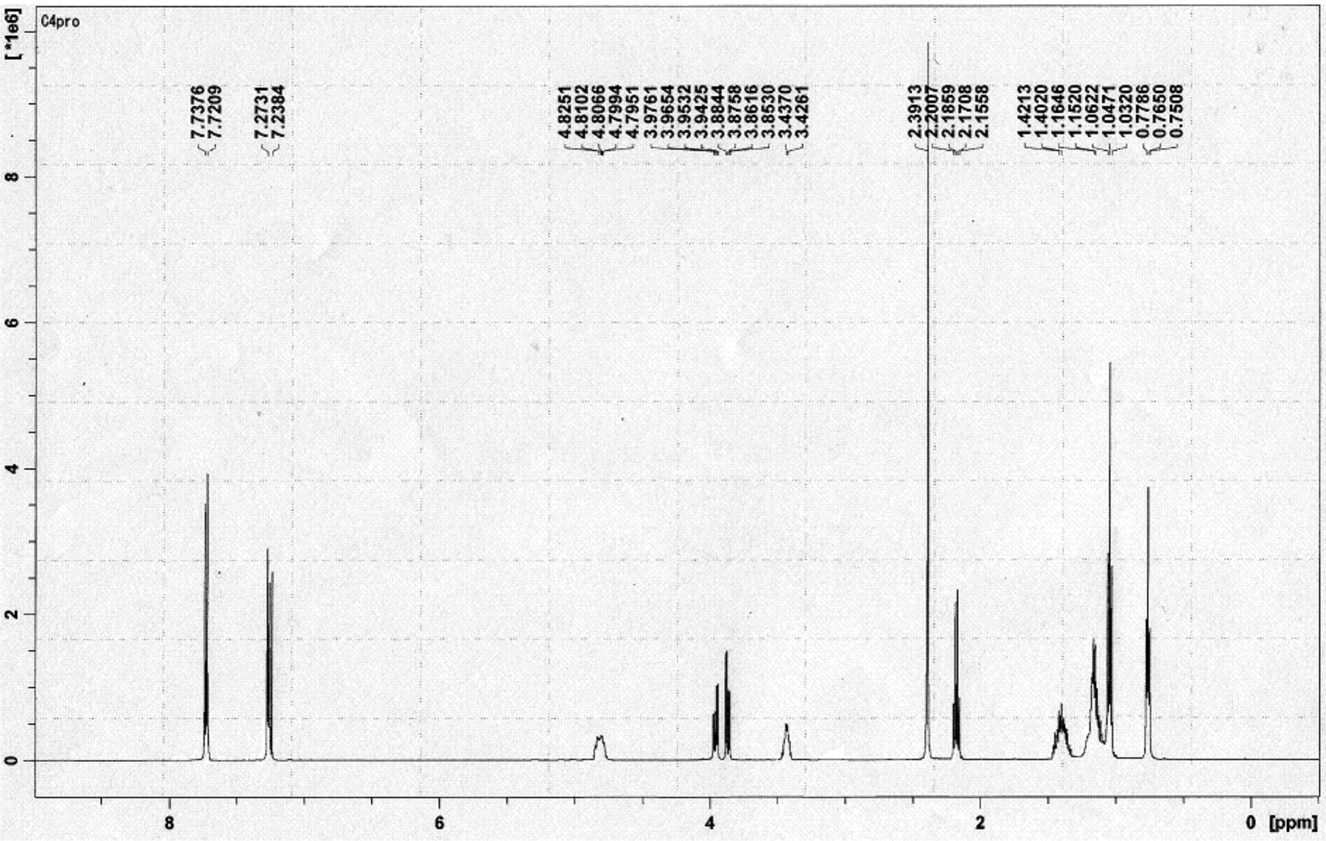

Figure S13. ^{1}H-NMR Spectrum of compound **2c′**.

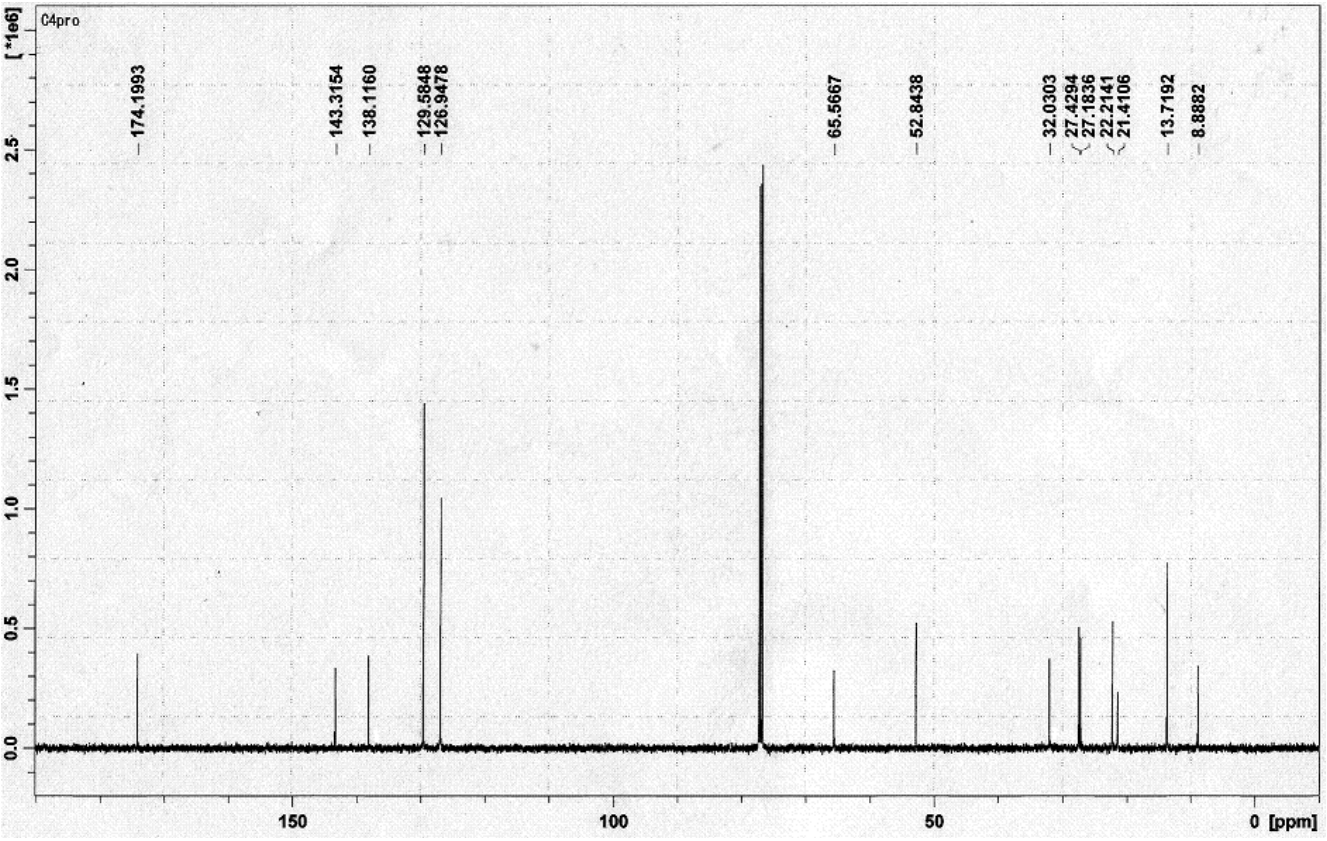

Figure S14. ^{13}C-NMR Spectrum of compound **2c′**.

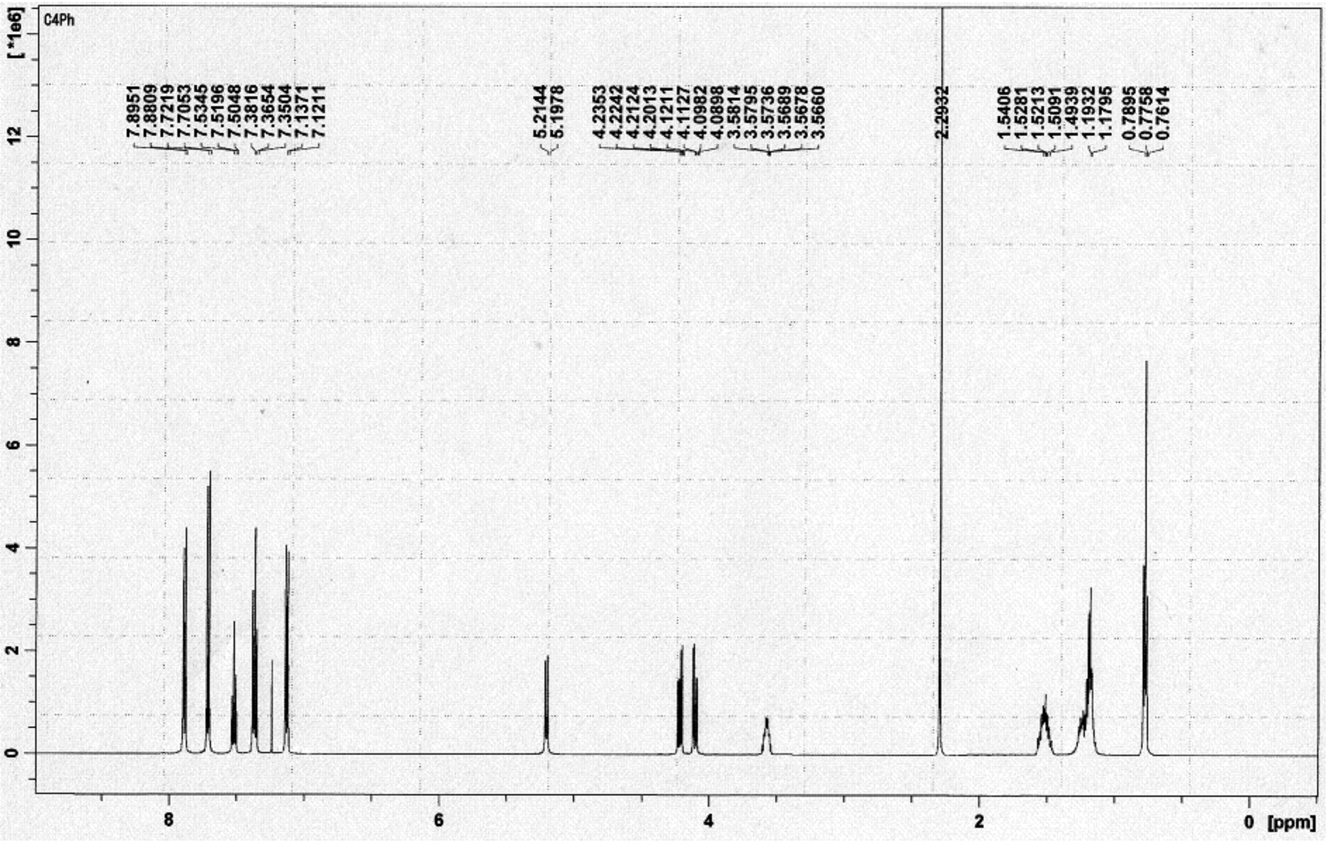

Figure S15. ^{1}H-NMR Spectrum of compound **2c″**.

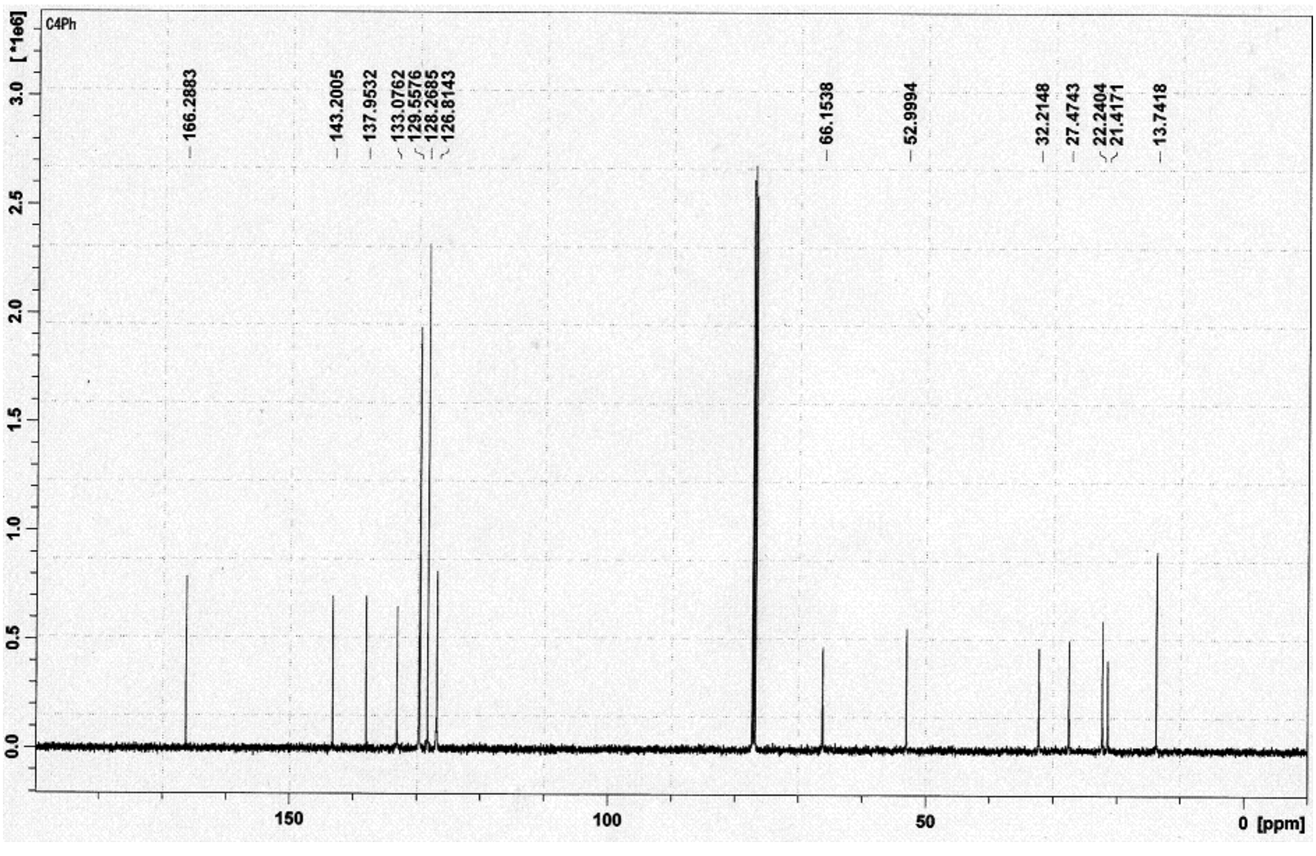

Figure S16. ^{1}H-NMR Spectrum of compound **2c″**.

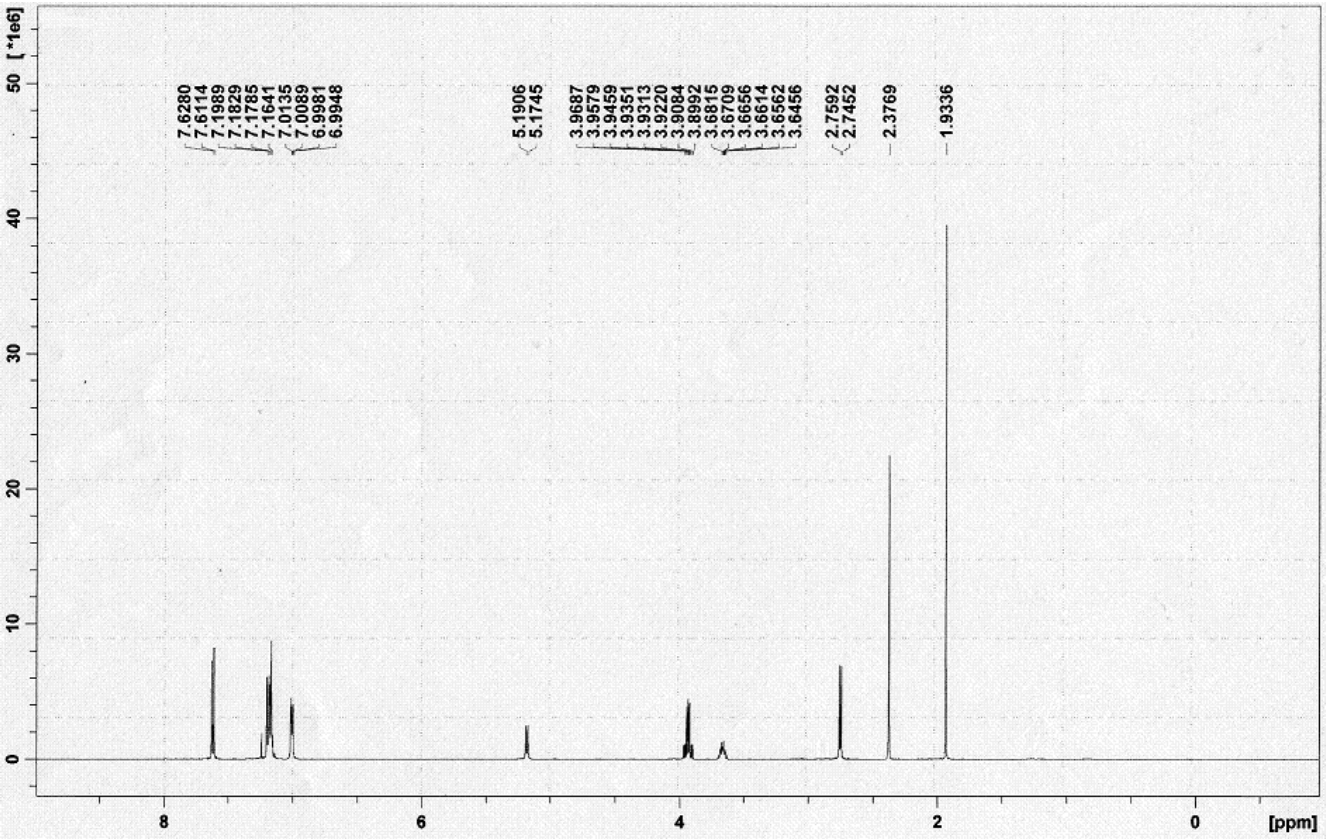

Figure S17. ^{1}H-NMR Spectrum of compound **2d**.

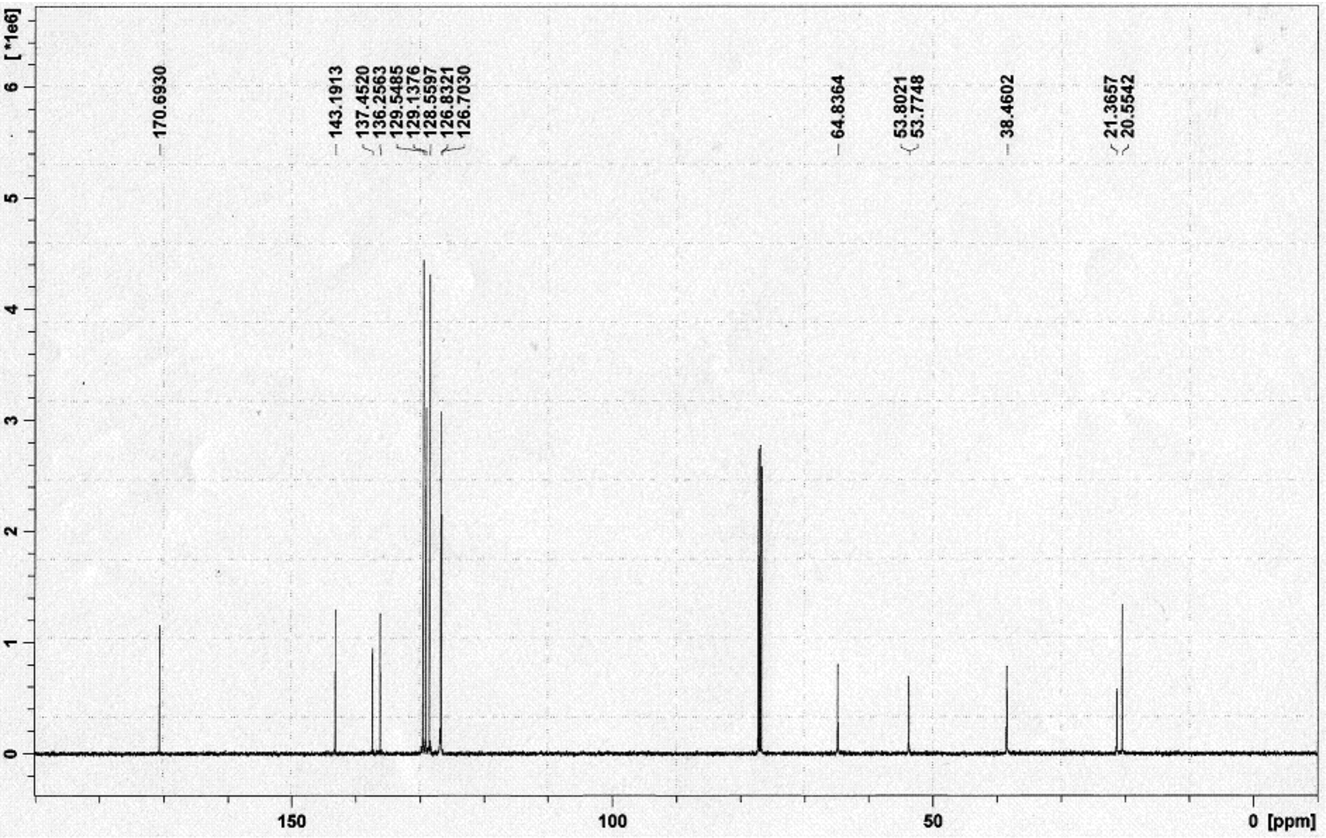

Figure S18. ^{13}C-NMR Spectrum of compound **2d**.

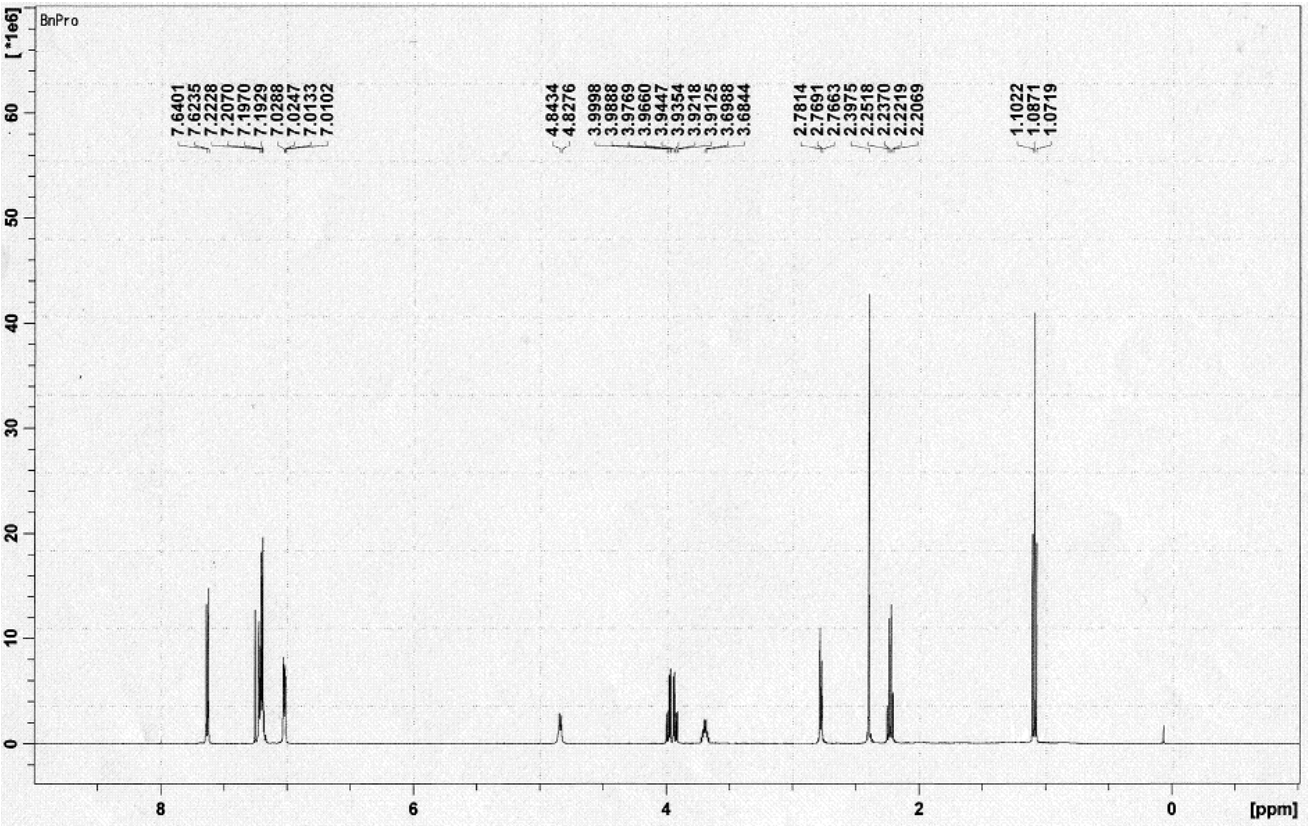

Figure S19. ^{1}H-NMR Spectrum of compound **2d′**.

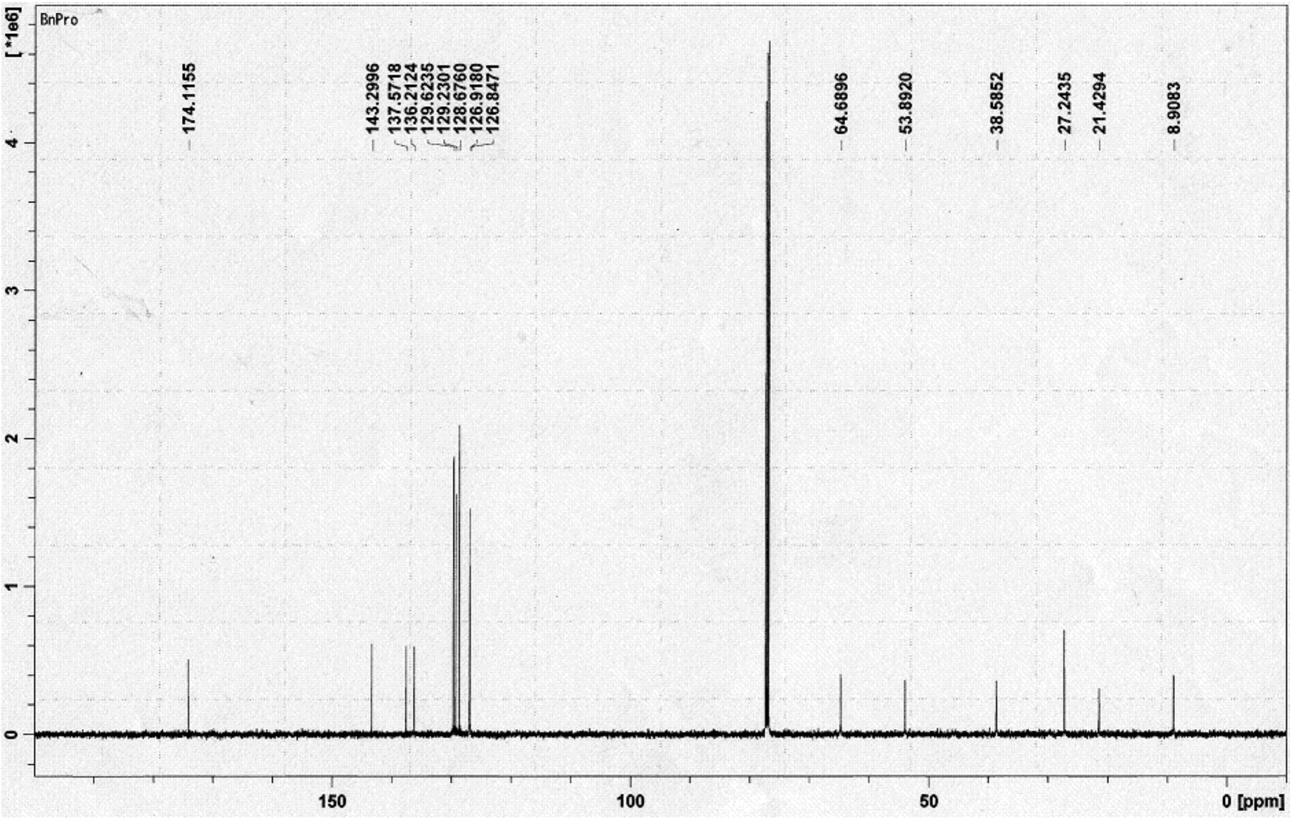

Figure S20. ^{13}C-NMR Spectrum of compound **2d′**.

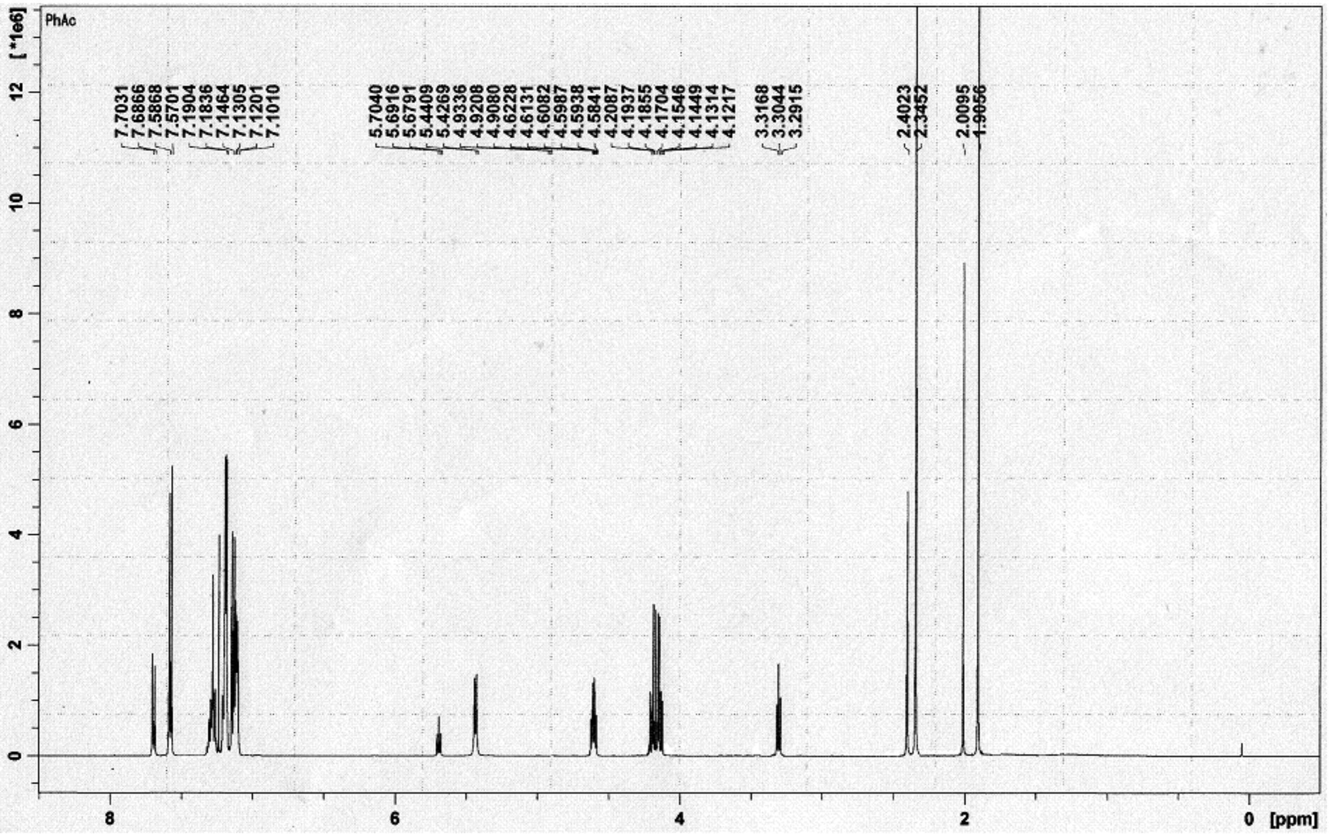

Figure S21. ^{1}H-NMR Spectrum of compound **2e**.

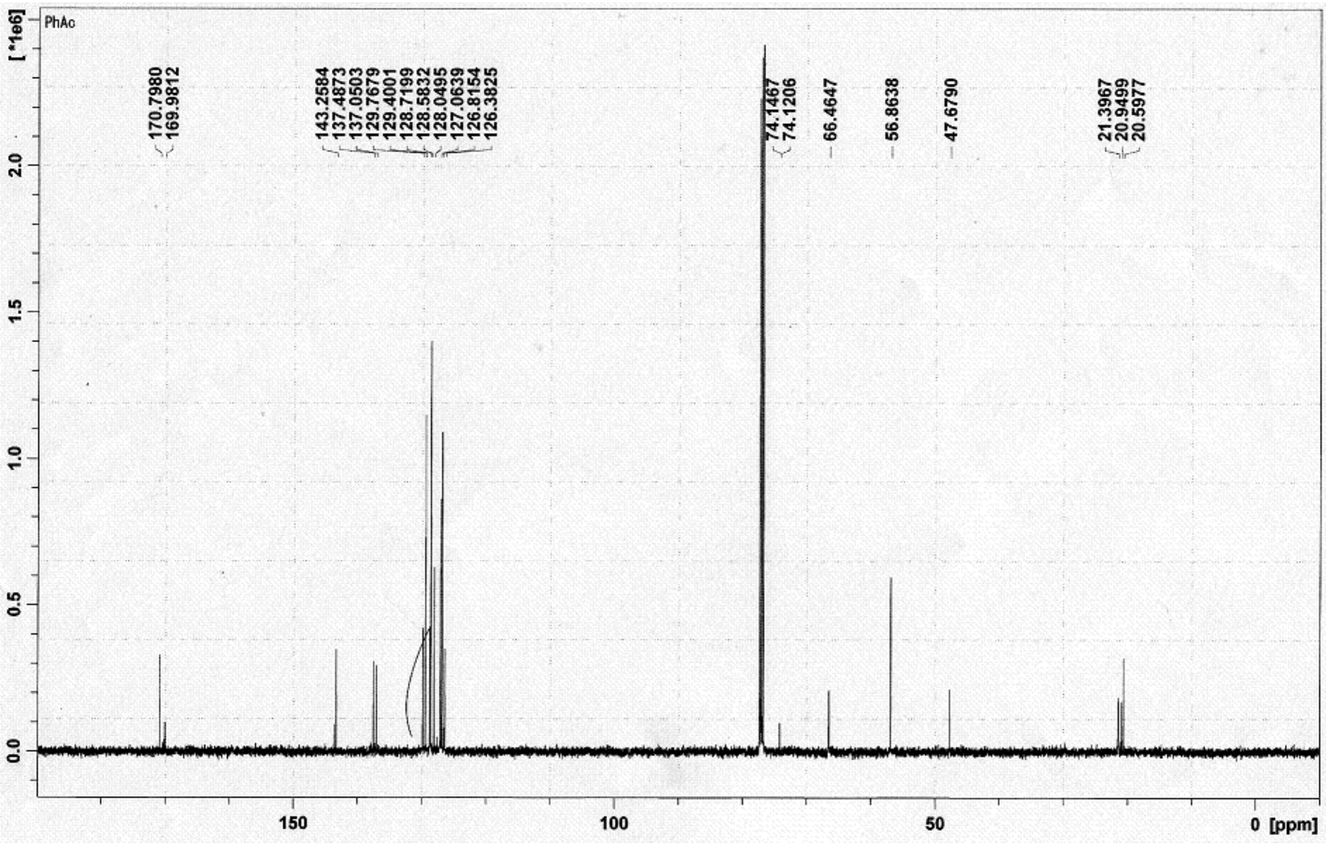

Figure S22. ^{13}C-NMR Spectrum of compound **2e**.

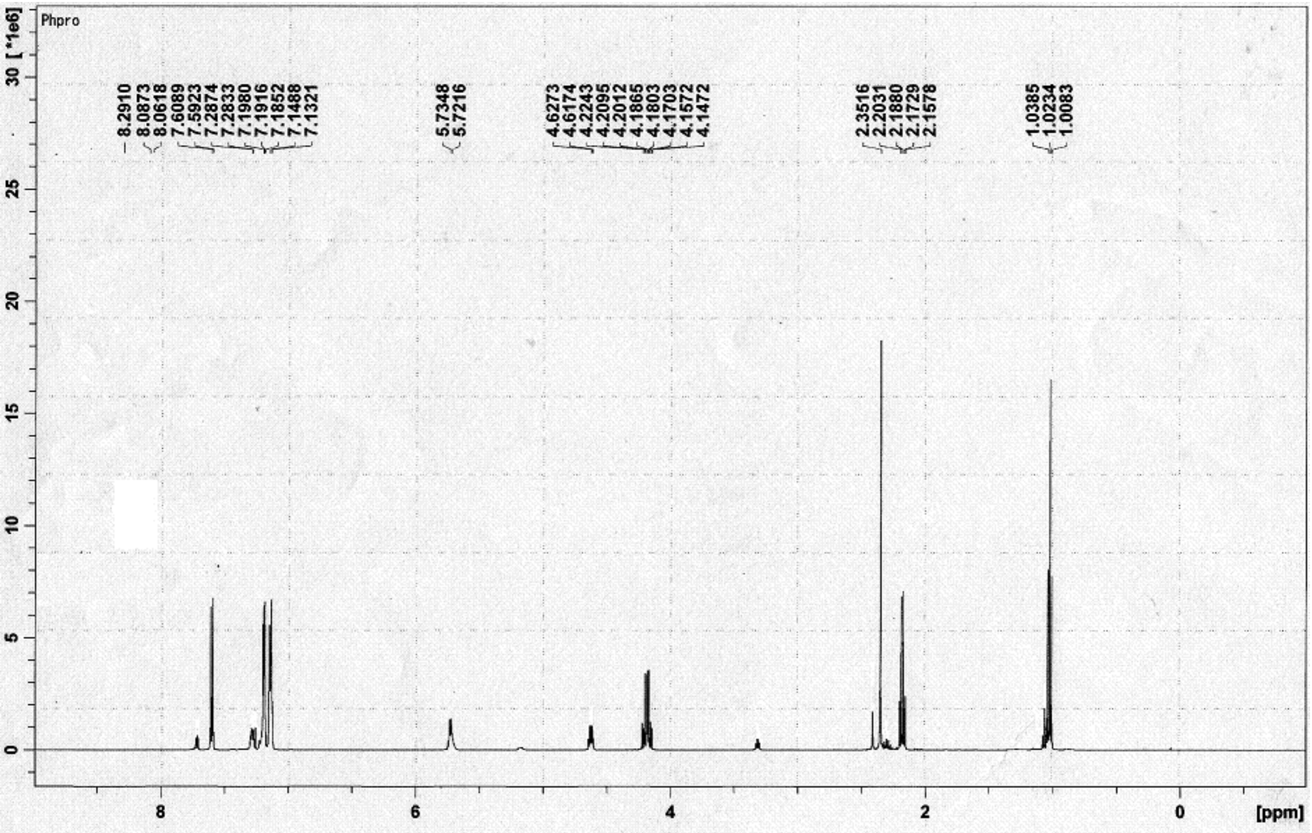

Figure S23. ^{1}H-NMR Spectrum of compound **2e′**.

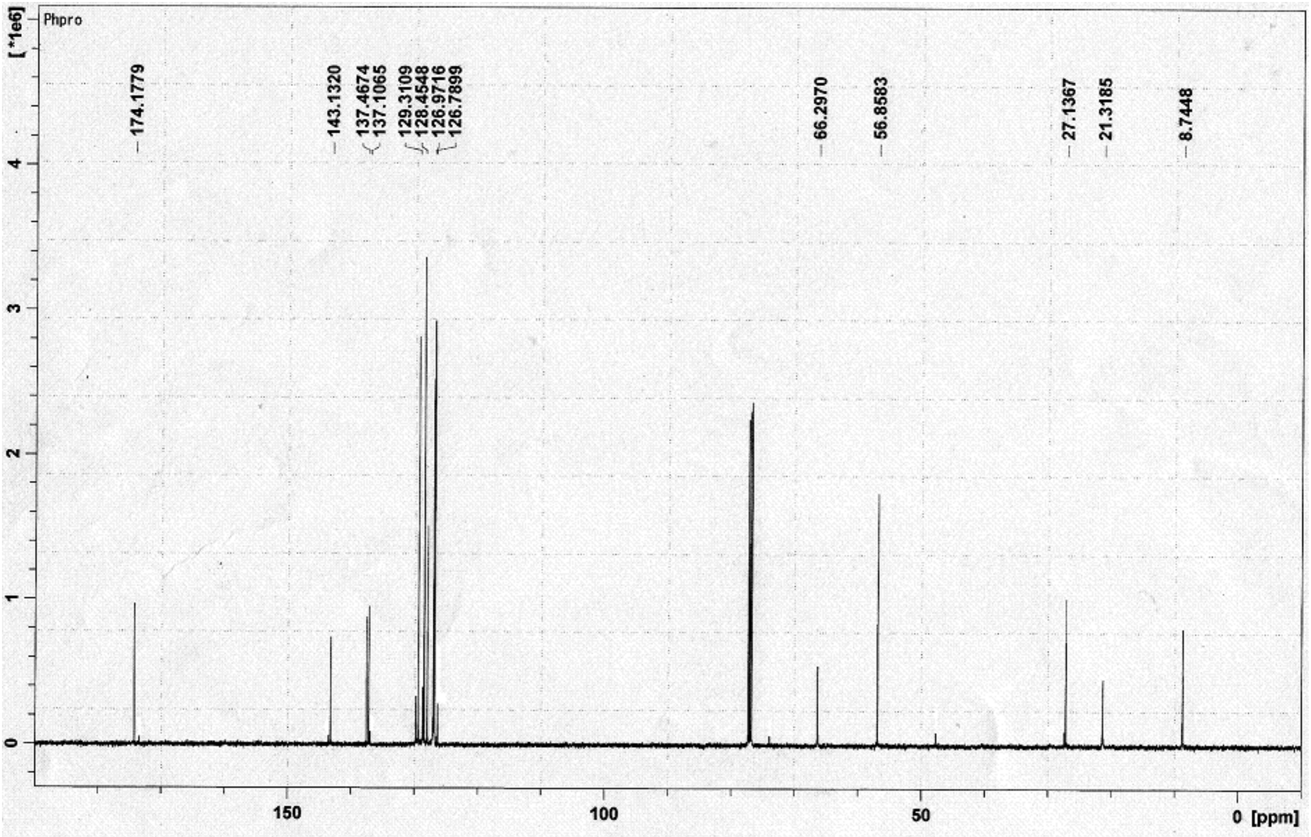

Figure S24. ^{13}C-NMR Spectrum of compound **2e′**.

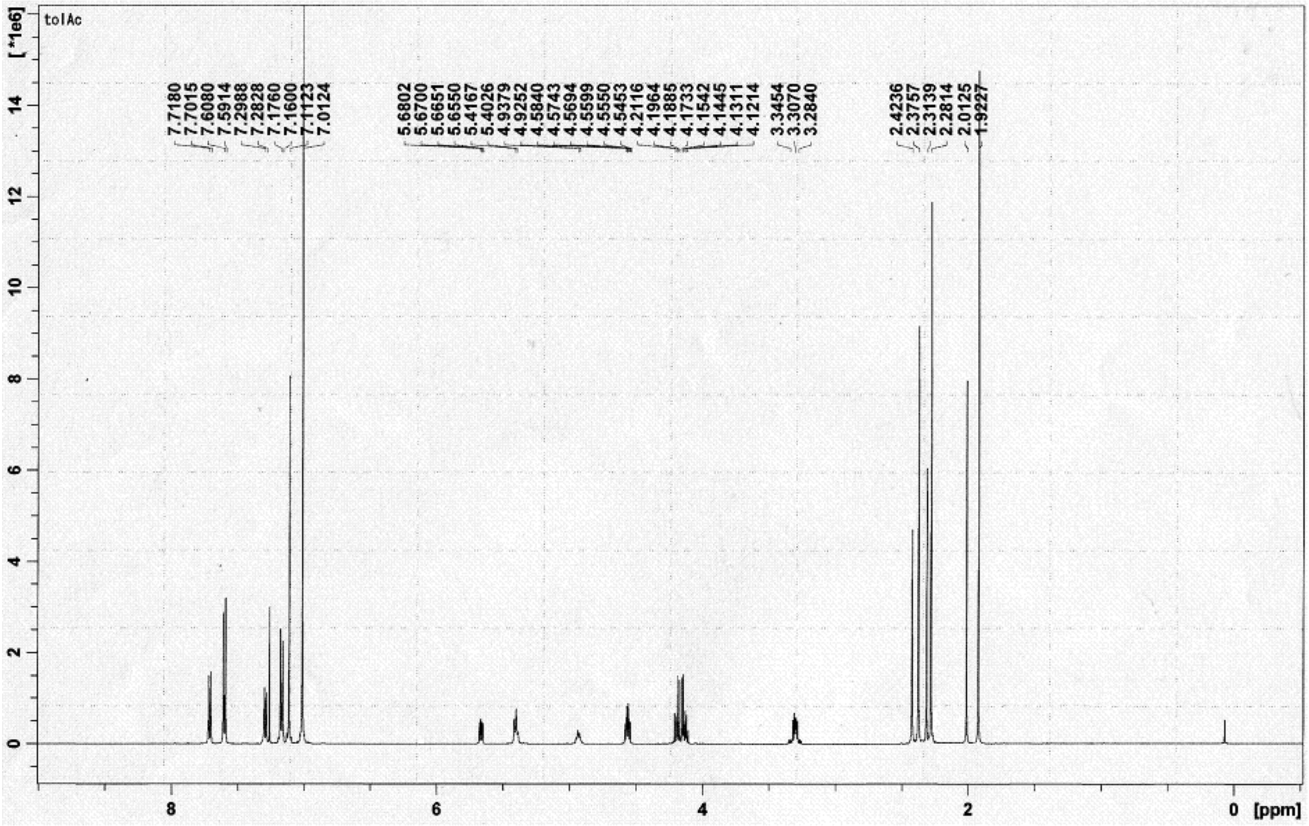

Figure S25. ^{1}H-NMR Spectrum of compound **2f**.

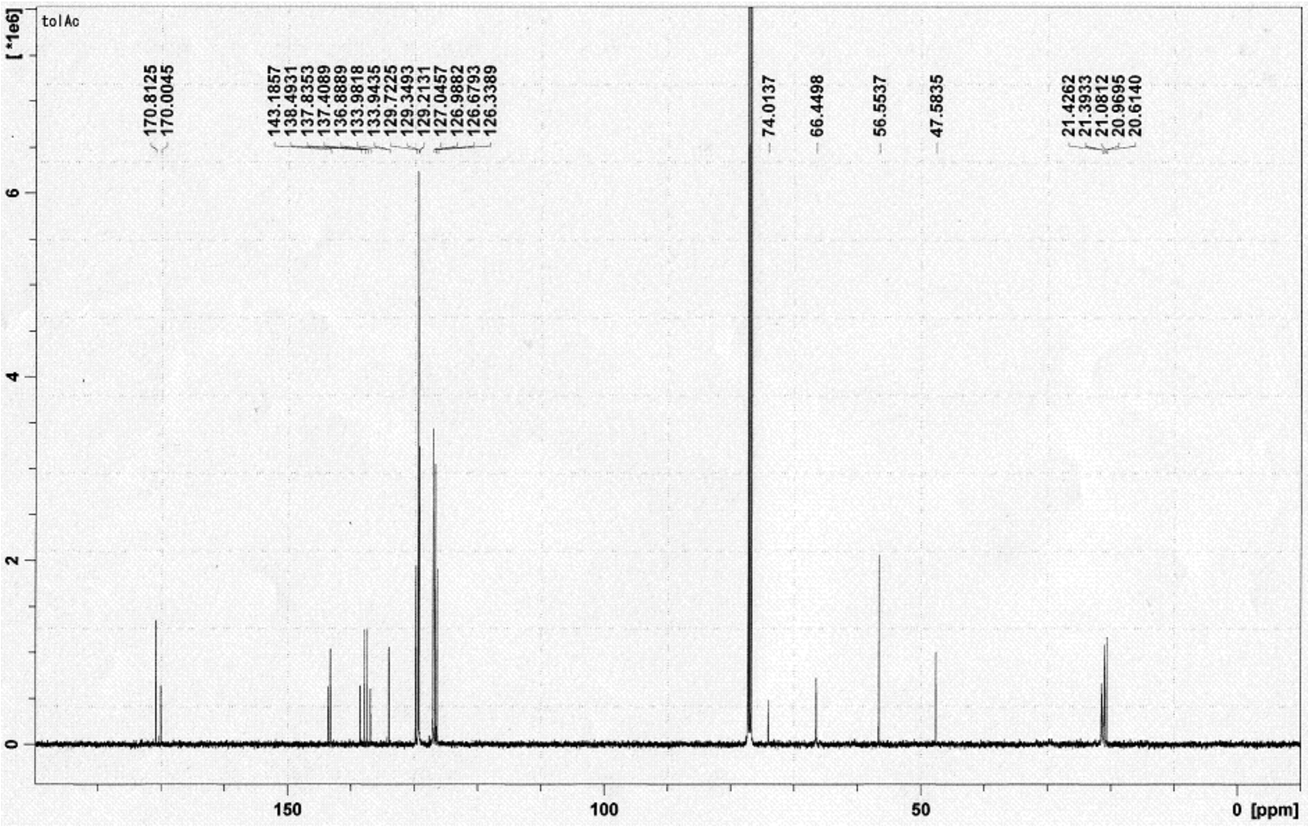

Figure S26. ^{13}C-NMR Spectrum of compound **2f**.

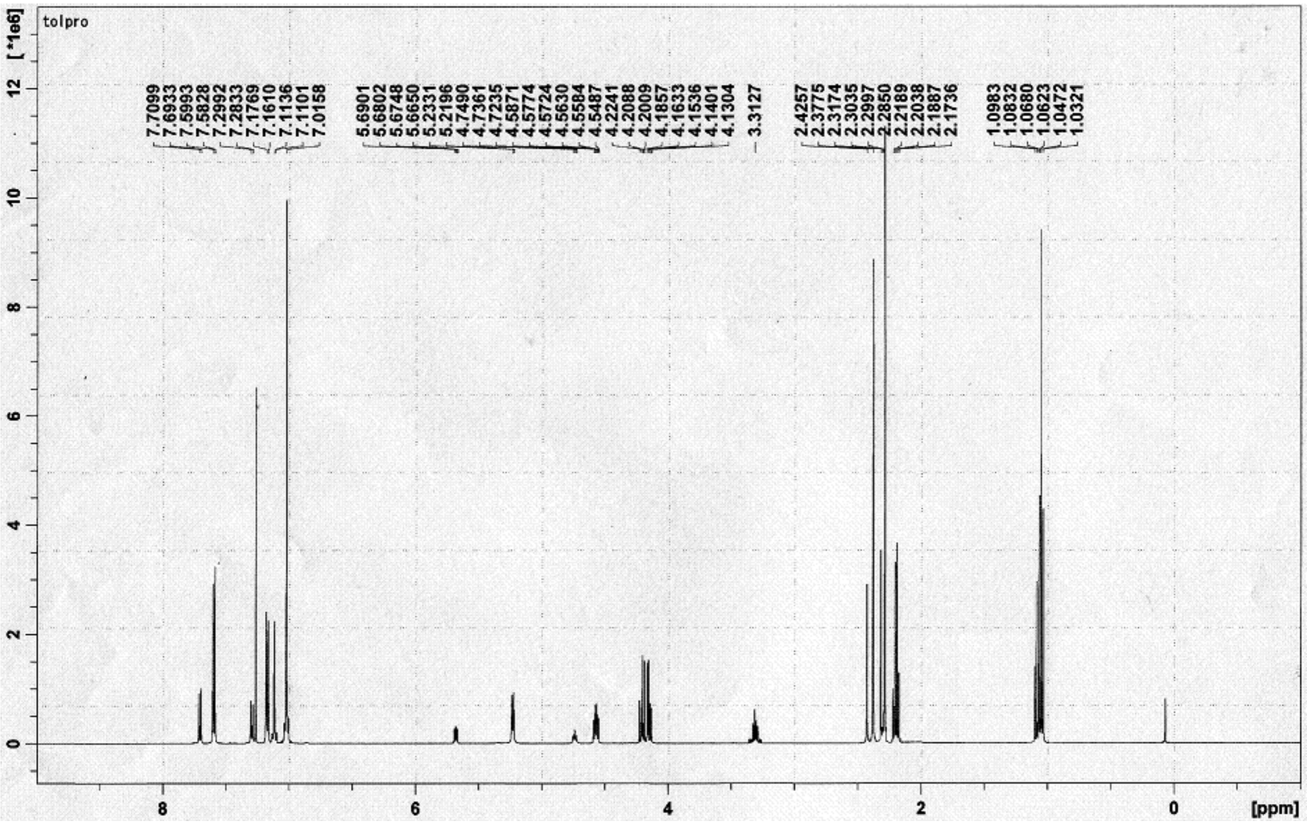

Figure S27. ^{1}H-NMR Spectrum of compound **2f′**.

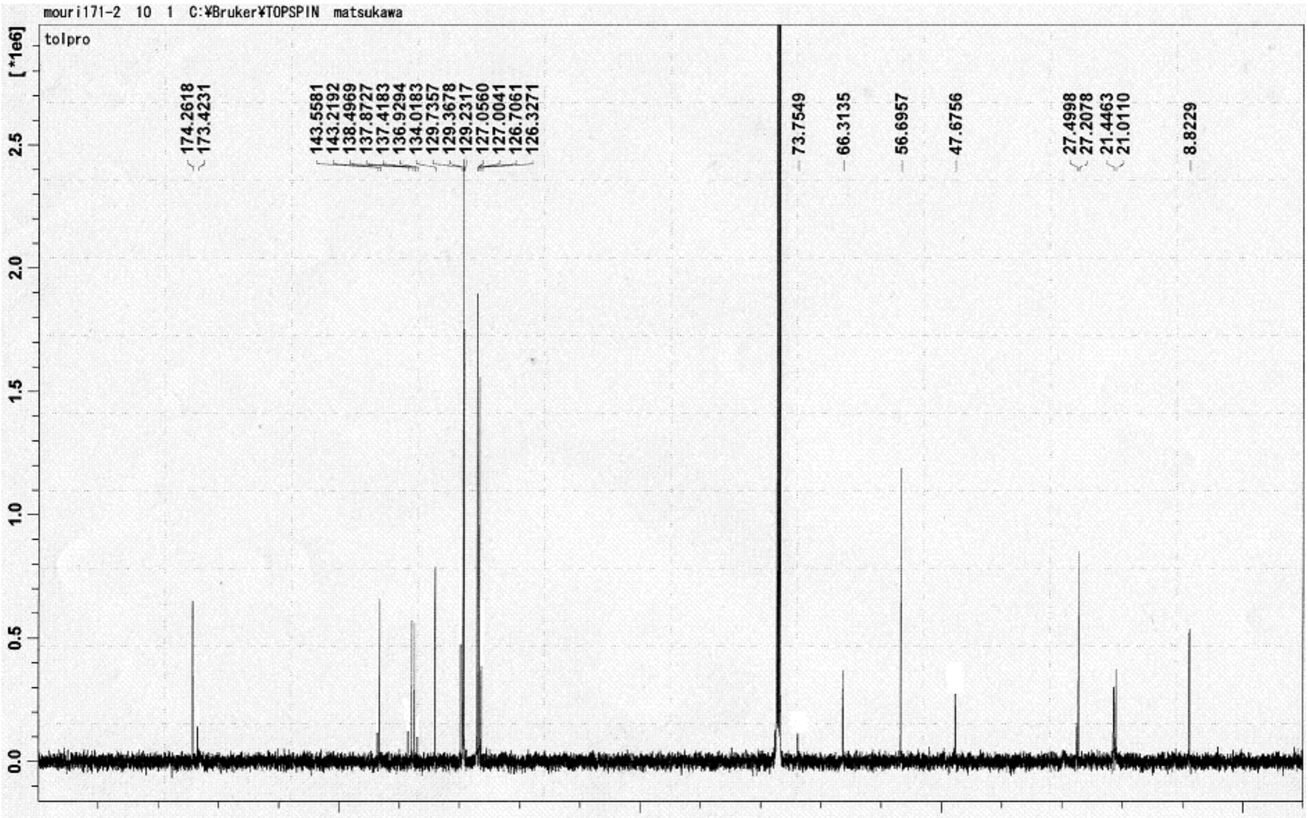

Figure S28. ^{13}C-NMR Spectrum of compound **2f′**.

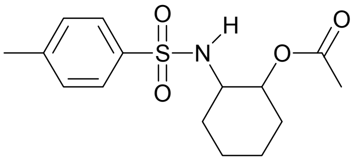

2a

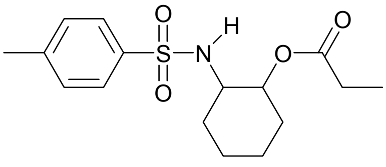

2a'

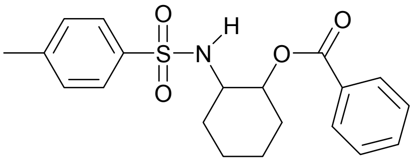

2a''

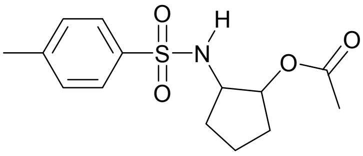

2b

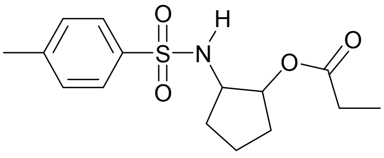

2b'

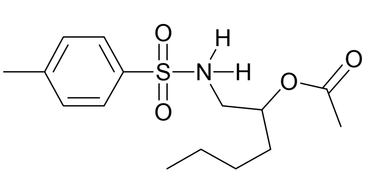

2c

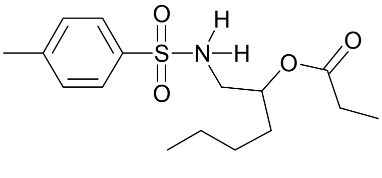

2c'

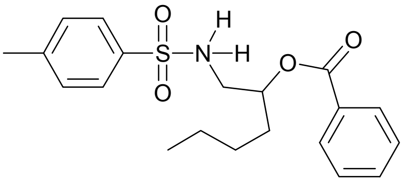

2c''

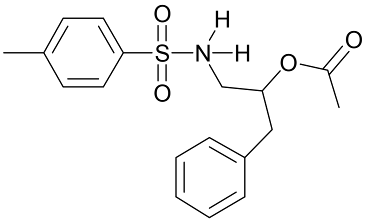

2d

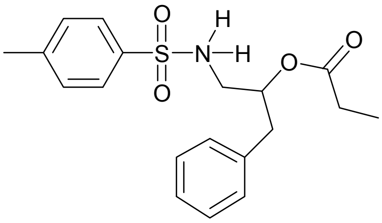

2d'

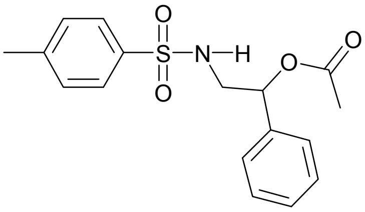

2e

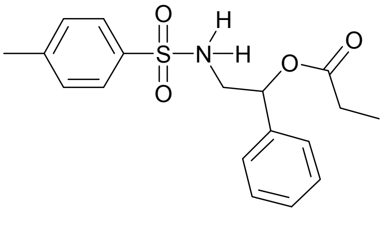

2e'

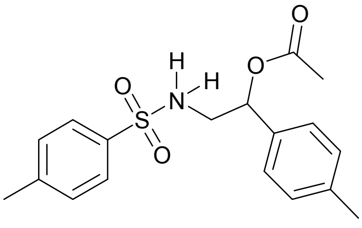

2f

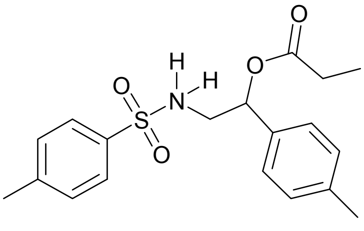

2f'

Figure S29. Structure of compounds **2a–2f**, **2a′–2f′**, **2a″**, and **2c″**.
